# Development and validation of a [^18^F]FDG PET/CT-based radiomics nomogram to predict the prognostic risk of pretreatment diffuse large B cell lymphoma patients

**DOI:** 10.1007/s00330-022-09301-5

**Published:** 2022-12-22

**Authors:** Mingshan Li, Hongyang Yao, Peng Zhang, Lingbo Zhang, Wei Liu, Zhiyun Jiang, Wei Li, Shu Zhao, Kezheng Wang

**Affiliations:** 1 PET-CT/MRI Department, Harbin Medical University Cancer Hospital, 150 Haping Road, Harbin, 150081 Heilongjiang China; 2 Urological Surgical Department, The 4th affiliated Hospital of Harbin Medical University, 37 Yiyuan Road, Harbin, 150001 Heilongjiang China; 3 Oral Department, The 2nd Affiliated Hospital of Harbin Medical University, 246 Xuefu Road, Harbin, 150001 Heilongjiang China; 4 Radiology Department, Harbin Medical University Cancer Hospital, 150 Haping Road, Harbin, 150081 Heilongjiang China; 5 Interventional Vascular Surgery Department, The 4th affiliated Hospital of Harbin Medical University, 37 Yiyuan Road, Harbin, 150001 Heilongjiang China; 6 Medical Oncology Department, Harbin Medical University Cancer Hospital, 150 Haping Road, Harbin, Heilongjiang 150081 China

**Keywords:** [^18^F]FDG PET/CT, Radiomics, Diffuse large B cell lymphoma, Progression-free survival, Nomogram

## Abstract

**Objective:**

In this study, based on PET/CT radiomics features, we developed and validated a nomogram to predict progression-free survival (PFS) for cases with diffuse large B cell lymphoma (DLBCL) treated with immunochemotherapy.

**Methods:**

This study retrospectively recruited 129 cases with DLBCL. Among them, PET/CT scans were conducted and baseline images were collected for radiomics features along with their clinicopathological features. Radiomics features related to recurrence were screened for survival analysis using univariate Cox regression analysis with *p* < 0.05. Next, a weighted Radiomics-score (Rad-score) was generated and independent risk factors were obtained from univariate and multivariate Cox regressions to build the nomogram. Furthermore, the nomogram was tested for their ability to predict PFS using time-dependent receiver operating characteristic (ROC) curves, calibration curves, and decision curve analysis (DCA).

**Results:**

Blood platelet, Rad-score, and gender were included in the nomogram as independent DLBCL risk factors for PFS. We found that the training cohort areas under the curve (AUCs) were 0.79, 0.84, and 0.88, and validation cohort AUCs were 0.67, 0.83, and 0.72, respectively. Further, the DCA and calibration curves confirmed the predictive nomogram’s clinical relevance.

**Conclusion:**

Using Rad-score, blood platelet, and gender of the DLBCL patients, a PET/CT radiomics-based nomogram was developed to guide cases’ recurrence risk assessment prior to treatment. The developed nomogram can help provide more appropriate treatment plans to the cases.

**Key Points:**

*• DLBCL cases can be classified into low- and high-risk groups using PET/CT radiomics based Rad-score.*

*• When combined with other clinical characteristics (gender and blood platelet count), Rad-score can be used to predict the outcome of the pretreatment of DLBCL cases with a certain degree of accuracy.*

*• A prognostic nomogram was established in this study in order to aid in assessing prognostic risk and providing more accurate treatment plans for DLBCL cases.*

**Supplementary Information:**

The online version contains supplementary material available at 10.1007/s00330-022-09301-5.

## Introduction

In addition to being one of the most common forms of non-Hodgkin lymphoma (NHL), diffuse large B cell lymphoma (DLBCL) also exhibits pronounced genetic, phenotypic, and clinical heterogeneity and a wide range of prognostic effects due to the high biological heterogeneity of DLBCL [1, 2]. Despite standard treatments such as immunochemotherapy of rituximab combined with cyclophosphamide, doxorubicin, vincristine, and prednisone, about 30–40% of cases suffer relapses or refractory disease with poor outcomes [3]. Therefore, one of the most important topics in the current diagnosis and treatment of lymphoma is to identify subtypes of such tumors based on their imaging and biological characteristics to reveal the biological risk and guide precise clinical treatments for the cases [4].

Considering the growing evidence of the heterogeneity of DLBCL, more clinical features, rather than a single clinicopathologic entity, need to be included to predict the prognosis [5]. Inflammation has long been associated with cancer biology [6], and it has been suggested that systemic inflammation plays a critical role in prognosis across a wide range of cancers [7–9].

The prognosis of lymphoma can also be improved with early detection and treatment, and it is recommended to evaluate DLBCL cases using ^18^F-fluorodeoxyglucose positron emission tomography/computed tomography ([^18^F]FDG PET/CT) before treatment [10]. Multiple studies have suggested that semiquantitative metabolic parameters of PET/CT images, including total lesion glycolysis (TLG), baseline metabolic tumor volume (MTV), and standardized uptake values (SUV), are independent prognostic factors for lymphoma, and they can be used to assist risk stratification, particularly among cases at high risk [11, 12].

More recently, radiomics has become an emerging concept as an intersection of computer science and medicine. Radiomics applies complex mathematical algorithms by deeper mining to obtain mass medical imaging data information from CT, MR, and PET [13]. Radiomics greatly combines the information from various medical images, and therefore the spatial and temporal heterogeneity of tumors can be observed in a comprehensive, noninvasive, and quantitative way [14, 15]. Many cancers, including lymphoma, have made significant progress in using radiomics features to evaluate efficacy and prognosis [16–18], but clinical guidelines incorporating these encouraging results have not yet been developed.

As a consequence, the objective of this study is to construct an effective clinical nomogram based on PET/CT radiomics signature (R-signature) and independent clinical prognostic markers for cases with DLBCL in order to predict their survival and guide individual treatment plans accordingly.

## Materials and methods

### DLBCL patient recruitment

From January 2013 to December 2018, we retrospectively enrolled cases with histologically confirmed DLBCL who had received PET/CT imaging scans at Harbin Medical University Cancer Hospital prior to treatment. There was no requirement for evidence of informed consent to be submitted since this study was retrospective, and the Institutional Review Board of the hospital approved the study. The following criteria were required for inclusion: (1) newly pathologically confirmed DLBCL; (2) no previous cancer history; (3) [^18^F]FDG PET/CT done less than two weeks prior to first treatment; (4) no antitumor therapy prior to scanning; and (5) availability of clinical and follow-up data. To determine their disease status, cases received anthracycline-based chemotherapy followed by CT or PET/CT scans with [^18^F]FDG. Following treatment for the first 2 years, follow-up assessments were performed every 3 months, then every 6 months, and the study’s primary end point is a patient’s PFS rate, which can be defined as the period between diagnosis and the date of the first relapse, progression, or death due to any cause. At the time of the last known follow-up, cases who had not experienced any events were censored.

### Administration of [^18^F]FDG and PET/CT acquisition

Fasting was required for 6–8 h before the examination, and blood glucose levels were controlled to be less than 11.1 mmol/L. Using Discovery 690 Elite (GE Healthcare), PET/CT scans were conducted on patients using [^18^F]FDG, which has a radiochemical purity of 98%. Approximately 1 h after IV [^18^F]FDG intravenous administration, a PET/CT scan was performed, covering the skull to the upper thigh anatomically. Cases firstly underwent spiral CT scanning at 120 kV, 140 mA, 1.25-mm pitch, and 3.75-mm layer thickness for 20–30 s. Next, PET imaging was conducted with a total of six to seven beds in 3D mode, for 2.5 min per bed, while the cases stayed in the same position. An ordered subset expectation maximization approach was used to iteratively reconstruct PET imaging data, and attenuation correction was carried out with CT data. The data were transmitted to a Xeleris™ Workstation (GE Healthcare) for PET/CT image fusion processing.

### VOI drawing and feature extraction

PET/CT images were transferred to an Advantage Workstation 4.5 (GE Healthcare) and reviewed by two experienced radiologists. Region of interest (ROI) was then extracted based on 41% of SUVmax as the threshold, and we calculated PET/CT metabolic parameters including MTV, SUVmax, SUVpeak, and TLG within ROI using PET VACR software [19]. Using Lugano classification, lesions were selected for analysis of texture features [20]. Lifex software (http://www.lifexsoft.org/.version 6.10) and ITK software (http://www.itksnap.org/.version 3.8.0) were then used to visualize PET and CT images of the target lesions. Additionally, texture features of the delineated target lesions were extracted using AK software 3.3 (GE Healthcare). In the analysis, 2074 radiomic features were extracted from CT and PET images, including the first order, shape, gray-level difference matrix, gray-level co-occurrence matrix, neighborhood gray tone difference matrix, gray-level size zone matrix, and gray-level run length matrix.

Randomly, 70% of the data were assigned to the training cohort and the rest of the data were assigned as the validation cohort. Texture features of the training cohort samples were analyzed by univariate Cox regression and then preliminarily screened for features related to the PFS (*p* < 0.05). The survival analysis was also further analyzed by Lasso-Cox regression to identify radiomic features associated with recurrence. Next, a Rad-score was constructed using the retained radiomic features and weighted by their coefficients obtained from linear combination calculations. Based on the median Rad-score obtained, cases were split into high-risk and low-risk groups, and their Kaplan-Meier survival curves were plotted in conjunction with the Rad-scores for each group. Log-rank tests were used to assess survival differences between groups, and ROC curves were used to assess the predictive value of PFS.

Univariate Cox regression was performed to analyze clinicopathological variables, PET/CT metabolic parameters, and Rad-score to determine significant risk factors (*p* < 0.05). Statistically significant variables were further analyzed with multivariate stepwise Cox regression to determine independent risk factors.

### Nomogram construction and performance assessment

Taking advantage of the radiomic features and independent risk factors, we further developed a nomogram for DLBCL patients based on PET/CT Rad-score. DCA, calibration curves, and ROC curves were used to assess the nomogram’s clinical utility and predictive capabilities.

### Statistical analyses

R software (version 3.4.2) was used throughout this study for statistical analysis. *p* < 0.05 indicated significant difference.

## Results

### Patients and groups

**Fig. 1:**
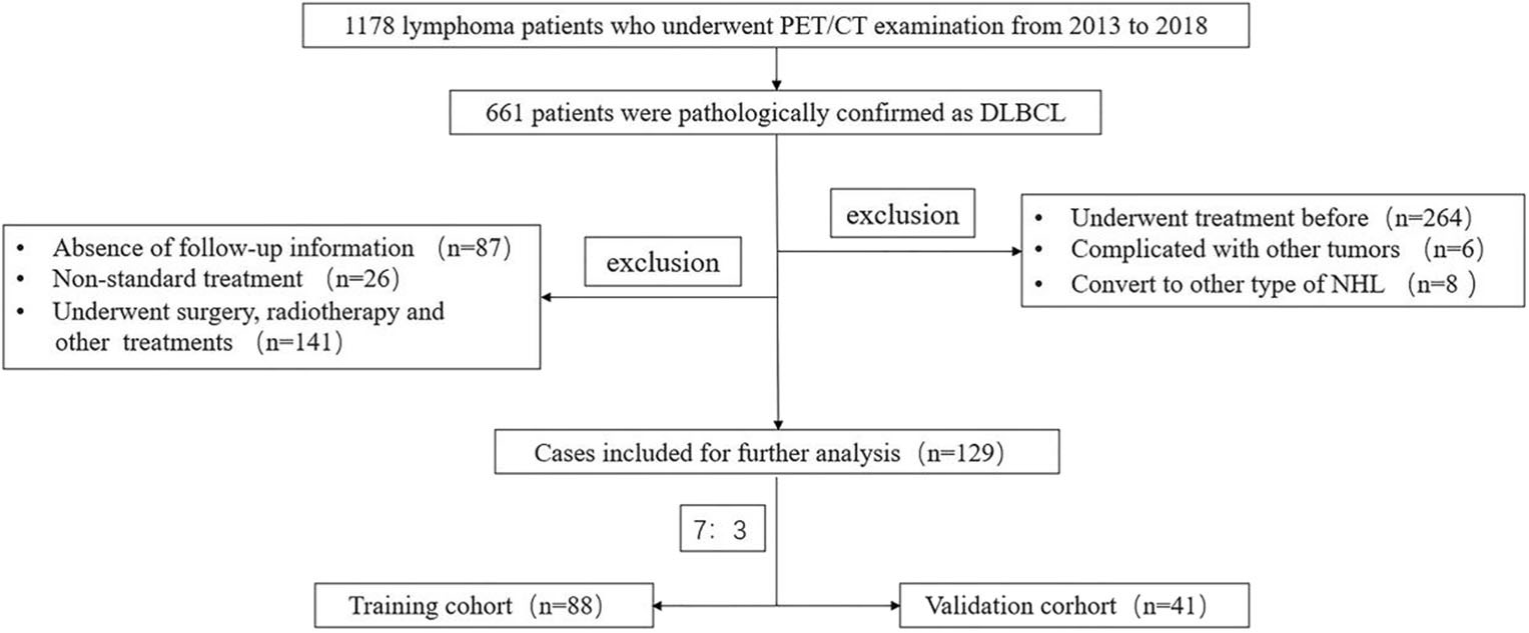
Flowchart of the enrolled patients according to inclusion and exclusion criteria

The study flowchart of the cases screening is presented in Fig. 1, and the statistical description of basic data is listed in Table 1. A total of 129 DLBCL cases were ultimately enrolled with 65 males and 64 females. Among the participants, the median age was 59 years old (range, 21 to 83 years old). The ratio of recurrence to non-recurrence during the follow-up period was 4:3. According to Table 1, no statistical differences were seen between the training cohort and validation cohort for any variables (*p* > 0.05).

**Table 1:** The baseline characteristics of patients with DLBCL in the training and validation cohorts

Characteristic	No. of patients			*p* value
	Overall (*n* = 129)	Training (*n* = 88)	Validation (*n* = 41)	
SUVmax	25.44 (18.02, 35.93)	27.45 ± 11.16	25.56 ± 10.31	0.348
SUVpeak	21.56 (14.66, 28.28)	22.74 ± 9.88	20.90 ± 9.08	0.3011
tMTV	93.71 (24.56, 205.89)	95.01 (25.04, 198.27)	51.30 (21.82, 235.70)	0.9094
TLG	1099.36 (300.30, 3415.80)	1121.73 (271.31, 3417.46)	983.96 (311.44, 2723.78)	0.8635
GCB				
0	94 (72.87%)	65 (73.86%)	29 (70.73%)	0.873
1	35 (27.13%)	23 (26.14%)	12 (29.27%)	
Ki67	70 (50, 80)	80 (57.5, 81.25)	70 (50, 80)	0.0958
Leukocyte	6.97 (5.53, 8.52)	7.09 (5.46, 8.62)	6.89 (5.79, 8.44)	0.9154
Neutrophil	4.59 (3.46, 5.92)	4.58 (3.46, 5.94)	4.63 (3.49, 5.51)	0.9677
Blood.platelet	242 (201, 311)	231 (199.25, 309.50)	265 (209, 321)	0.3639
Lymphocyte	1.52 (1.06, 2.07)	1.51 (1.10, 2.07)	1.52 (1.00, 1.97)	0.777
Hemoglobin	128.67 ± 18.49	128.85 ± 17.81	128.26 ± 20.10	0.8727
Monocyte	0.56 (0.43, 0.82)	0.57 (0.43, 0.84)	0.56 (0.42, 0.77)	0.9617
Age	59 (51,68)	62 (53, 8.25)	56 (51, 66)	0.1587
Gender				
Male	65 (50.39%)	45 (51.14%)	20 (48.78%)	0.9521
Female	64 (49.61%)	43 (48.86%)	21 (51.22%)	
ECOG	1 (0, 2)	1 (0.75, 2)	1 (0, 2)	0.9678
B.sympotom				
0	101 (78.29%)	69 (78.41%)	32 (78.05%)	1
1	28 (21.71%)	19 (21.59%)	9 (21.95%)	
Ann.Arbor				
1	18 (13.95%)	16 (18.18%)	2 (4.88%)	0.1021
2	42 (32.56%)	25 (28.41%)	17 (41.46%)	
3	25 (19.38%)	15 (17.05%)	10 (24.39%)	
4	44 (34.11%)	32 (36.36%)	12 (29.27%)	
IPI	2 (1, 3)	2 (1, 3)	2 (1, 3)	0.6996
LDH	236 (180, 379)	242 (176.25, 381.50)	236 (187, 370)	0.7119
LMR	2.71 (1.60, 4.22)	2.67 (1.63, 4.36)	2.89 (1.60, 3.86)	0.9335
NLR	3.10 (2.05, 4.36)	3.13 (1.99, 4.40)	2.89 (2.13, 3.86)	0.8694
PLR	169.42 (118.52, 251.14)	172.57 (109.94, 241.85)	162.09 (129.71, 251.94)	0.5901
Recurrence				
0	57 (44.19%)	40 (45.45%)	17 (41.46%)	0.8145
1	72 (55.81%)	48 (54.55%)	24 (58.54%)	

### Features selection and Rad-score construction

We first screened the variables associated with PFS using a univariate Cox regression based on radiomics features from the training cohort. Using *p* < 0.05 as the statistical significance, 731 features were obtained (Supplementary Table 1). In addition, to screen out features with non-zero coefficients, Lasso-Cox regression was performed, and Rad-score equations were constructed based on their coefficients as follows:

- Rad−score=original_shape_Elongation.PET × (−0.27781)
- +wavelet.HLH_ngtdm_Coarseness.PET × (−0.0758)
- +original_shape_Elongation.CT × (−0.04707)
- +original_shape_MajorAxisLength.CT × 0.001085
- +original_shape_Maximum2DDiameterRow.CT × 0.000138

### Radiomics features assessment

**Fig. 2:**
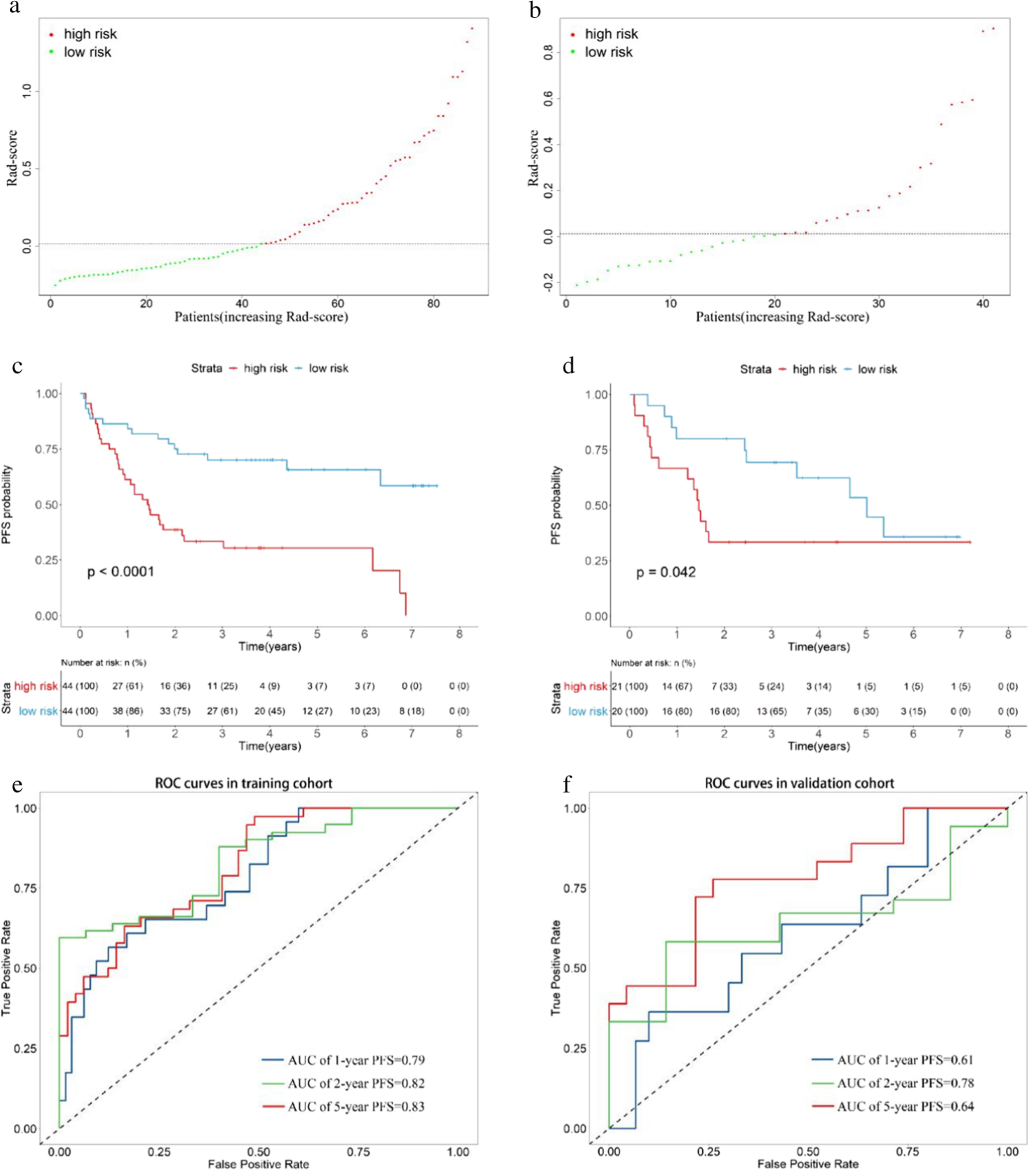
Rad-score analysis of patients in the training and validation cohorts. Risk chart of the training cohort (**a**) and validation cohort (**b**). Rad-score measured by K-M survival curves of the training cohort (**c**) and validation cohort (**d**), the log-rank test was used to calculate *p* values, *p* < 0.05 and the differences were significant, both cohorts were divided into high-risk and low-risk groups. The ROC curves of the training cohort (**e**) and validation cohort (**f**) to predict the 1-PFS, 2-PFS, and 5-PFS

The training cohort was divided into two groups based on the median Rad-score as shown in Fig. 2a and b. The higher the Rad-score, the greater the risk and the more likely a recurrence. Figure 2c indicates the K-M survival curves of cases from two groups of the training cohort and shows a statistically significant difference in recurrence risk (*p* < 0.0001). As shown in Fig. 2d, similar results were observed. The ROC curves plotted in Fig. 2e show the prediction of the recurrence at 1, 2, and 5 years with Rad-scores, and AUC values of 0.79, 0.82, and 0.83, respectively. Validation cohort results are shown in Fig. 2f, with AUC values of 0.61, 0.78, and 0.64, respectively.

### Nomogram construction

**Fig. 3:**
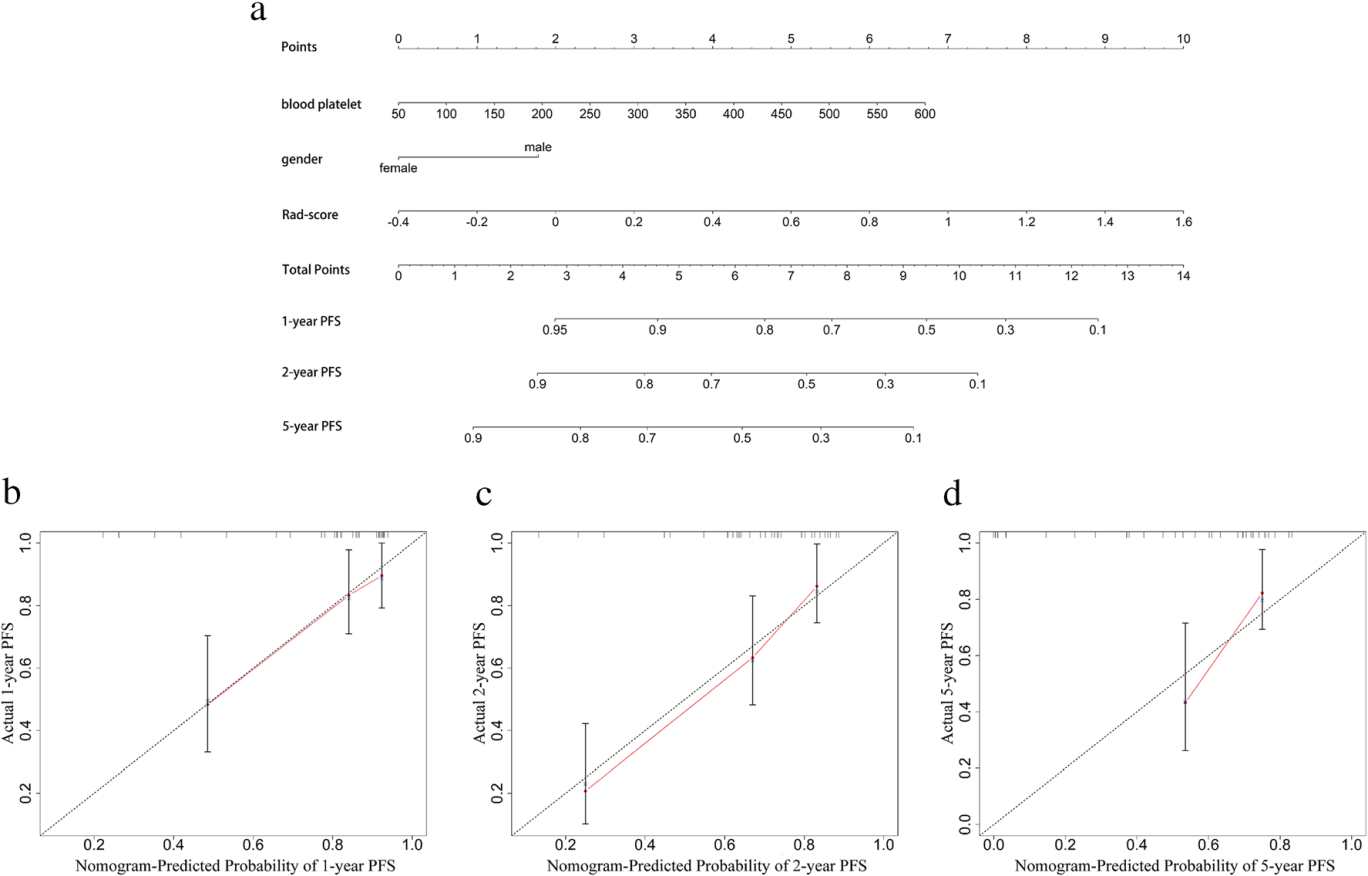
The visualization of PFS survival models based on pre-treatment PET-CT radiomics signatures combined with clinical characteristics and the calibration curves. **a** The nomogram combined with the Rad-score and the independent clinical risk factors, including gender and blood platelet, to predict the risk of PFS at 1, 2, and 5 years. **b**–**d** To predict the PFS of DLBCL using the nomogram and calibrate for the predictive model. The diagonal dotted line represents the ideal state, and the solid red line represents the actual predictive value: the closer it is to the diagonal dotted line, the better the predictive power

Using clinical variables, metabolic parameters, and Rad-scores for the training cohort samples, univariate Cox regression was carried out and the result is shown in Table 2. In the following multivariate stepwise Cox regression analysis, clinical variables that were statistically significant in the univariate analysis were included. The results are shown in Table 3. Blood platelet count, gender, and Rad-score were found to be statistically significant independent prognostic factors for predicting PFS using multivariate analysis, and a nomogram was built thereafter to predict the individualized PFS (Fig. 3a).

**Table 2:** Univariate Cox regression on clinical variables of the training cohort samples

Variables	HR	HR (95% CI)	*p*
SUVmax	1.007893	0.9840812–1.03228	0.519
H_SUVpeak	1.009681	0.983314–1.036756	0.475
tMTV	1.001717	1.000734–1.0027	0.0006
TLG	1.000102	1.000031–1.000173	0.005
GCB	1.516	0.8229–2.792	0.182
Ki67	0.992	0.9752–1.009	0.353
Leukocyte	1.068	0.973–1.171	0.167
Neutrophil	1.099	0.9892–1.222	0.0786
Blood.platelet	1.003	1.001–1.006	0.0157
Lymphocyte	0.7266	0.4998–1.056	0.0942
Hemoglobin	0.9956	0.9799–1.012	0.588
Monocyte	3.558	1.677–7.55	0.0009
Age	1.014	0.9918–1.037	0.214
Gender	1.861	1.036 –3.342	0.0377
ECOG	1.207	0.8418–1.731	0.306
B.sympotom	1.292	0.6713–2.487	0.443
Ann.Arbor	1.587	1.22–2.064	0.0006
IPI	1.459	1.152–1.849	0.0017
LDH	1.001	1.001–1.002	< 0.0001
LMR	0.7183	0.5924–0.8711	0.0008
NLR	1.207	1.079–1.351	0.0011
PLR	1.004	1.002–1.006	0.0009
Rad-score	7.222	3.762–13.87	< 0.0001

**Table 3:** Multivariate Cox regression on statistically significant clinical variables in the univariate analysis

Variables	HR	HR (95% CI)	*p*
Blood platelet	1.0048	1.0016–1.0080	0.0039
Gender	2.0373	1.0849–3.8260	0.0269
Rad-score	9.4649	3.3503–26.7390	< 0.0001

### Model assessment

The degree of fit between the cases’ outcomes and the nomogram prediction was calculated after calibration curves were plotted. Figure 3b, c and d show the calibration curves of the 1-, 2-, and 5-year PFS of this nomogram. A model with a higher accuracy will be closer to a diagonal dotted line, demonstrating excellent agreement between predictions and clinical observations.

**Fig. 4:**
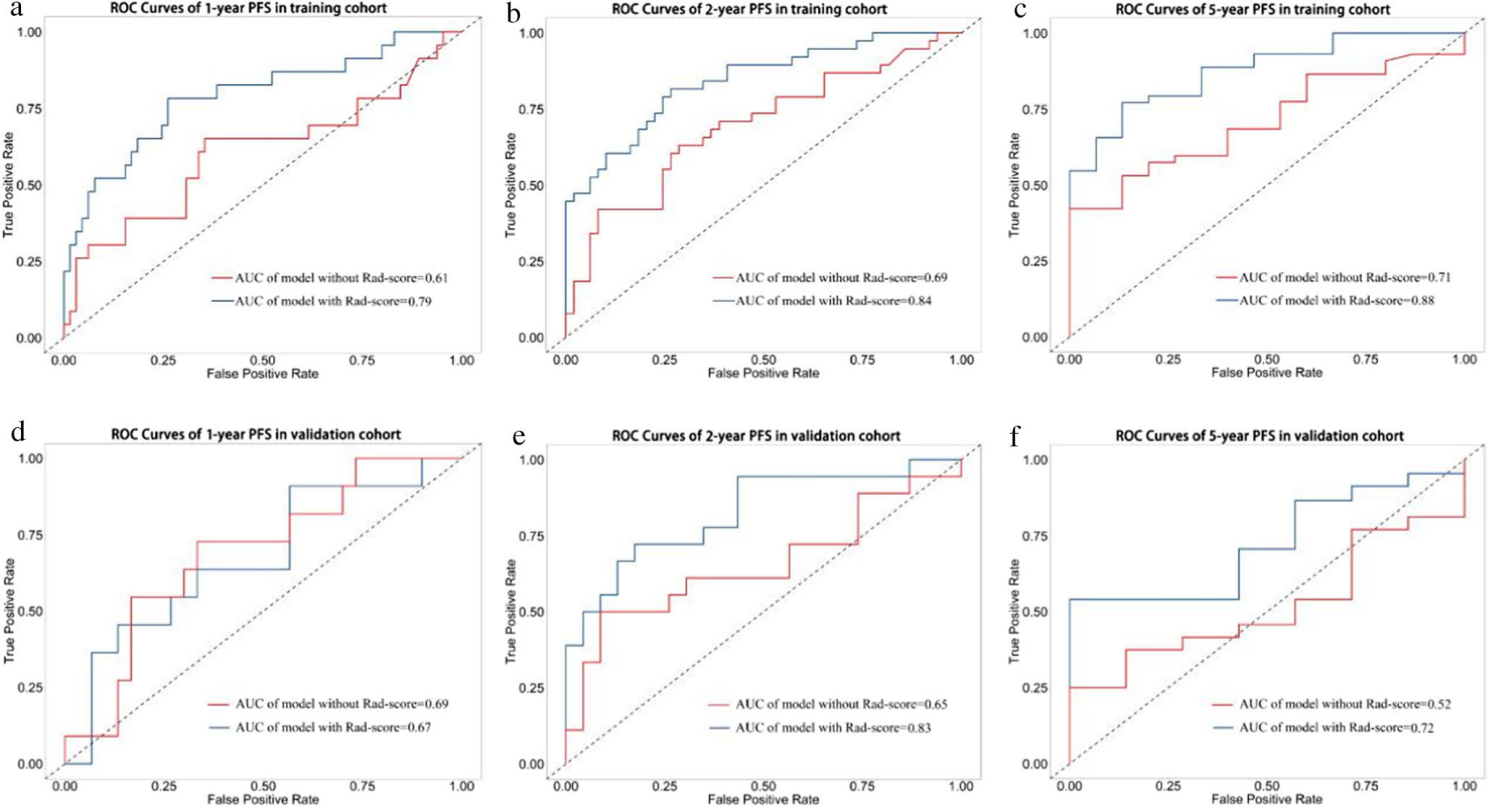
The ROC curves of the models for evaluating the PFS in the training and validation cohorts. **a**–**c** The ROC curves of the comparison between the model with or without Rad-score to predict the 1-, 2-, and 5-year PFS in the training cohort. **d**–**f** The ROC curves of the comparison between the model with or without Rad-score to predict the 1-, 2-, and 5-year PFS in the validation cohort. It was found that the model with Rad-score was better than the model without Rad-score in predicting PFS

ROC curves for the prediction model were also configured. Figure 4 represents the time-dependent ROC curves of the training (Fig. 4a, b and c) and validation (Fig. 4d, e and f) cohorts with or without Rad-score parameters at 1-, 2-, and 5-year PFS, respectively. Results showed that clinically independent prognostic factors with Rad-scores significantly improved the predictive accuracy and the clinical diagnostic ability of the model (training cohort: 1-year PFS AUC 0.79 vs. 0.61; 2-year PFS AUC 0.84 vs. 0.69; 5-year PFS AUC 0.88 vs. 0.71; validation cohort: 1-year PFS AUC 0.67 vs. 0.69; 2-year PFS AUC 0.83 vs. 0.65; and 5-year PFS AUC 0.72 vs. 0.52).

**Fig. 5:**
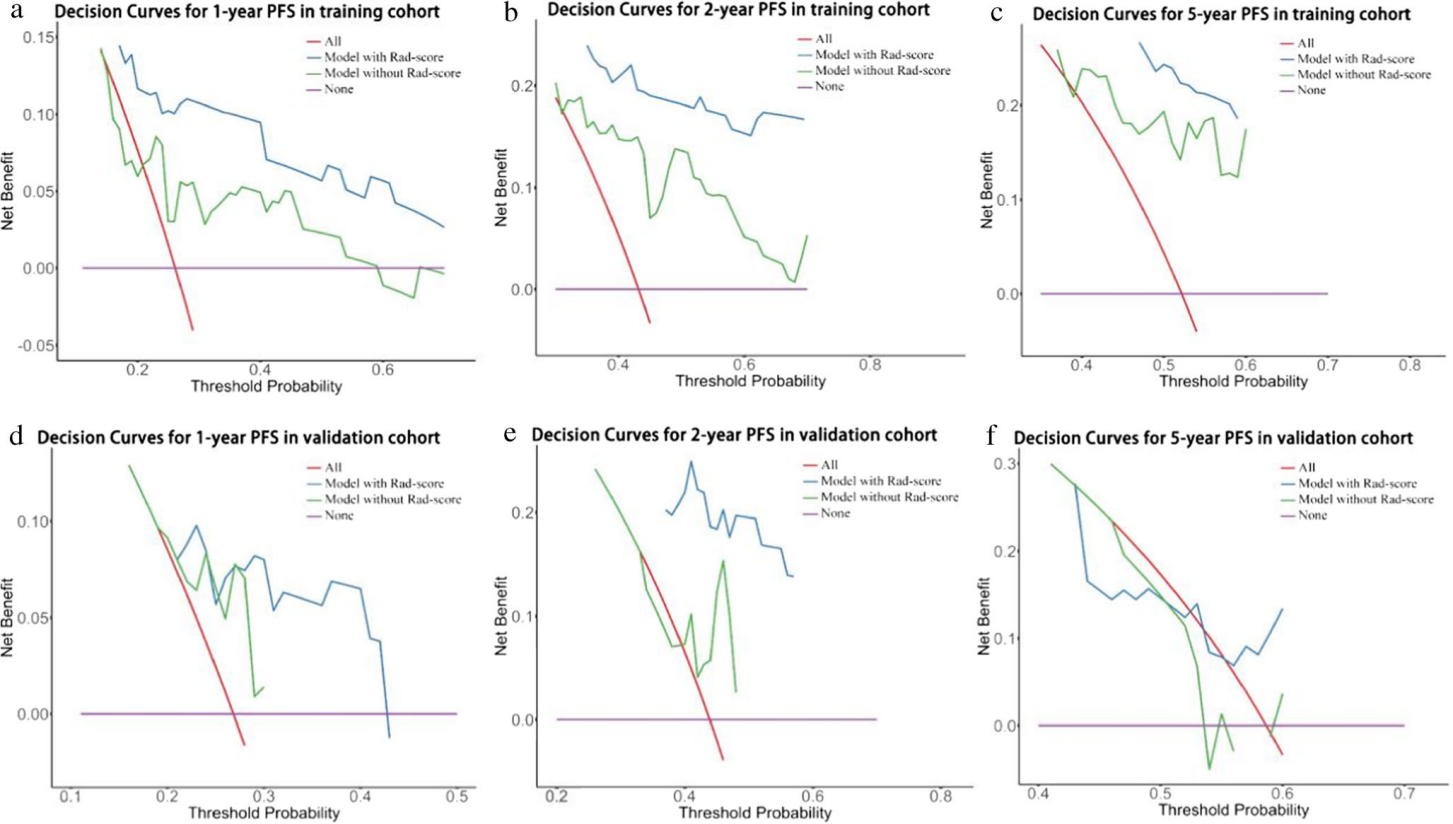
The DCA curves of the models in training and validation cohorts. **a**–**c** The DCA curves of the comparison between the model with or without Rad-score to predict the 1-, 2-, and 5-year PFS in the training cohort. **d**–**f** The DCA curves of the comparison between the model with or without Rad-score to predict the 1-, 2-, and 5-year PFS in the validation cohort. DCA curves showed that the model with Rad-score benefits for patients in the prediction of PFS at 1, 2, and 5 years

A Rad-score was not included in the validation and training cohorts of the clinical model to demonstrate the Rad-score’s contribution. Figure 5 displays the decision curves with or without Rad-scores of the training cohorts (Fig. 5a, b and c) and validation (Fig. 5d, e and f) cohorts, respectively. The results showed that the clinical prediction benefit was better after combining Rad-scores, indicating that it has a certain clinical application value.

### Independent verification

To further verify the value of the prediction model in practical application, we included another 32 cases newly diagnosed with DLBCL from January 2019 to December 2020 from the same hospital for independent validation. The results confirmed that 23 cases who received standard treatment had the same status as the nomogram predicted, with an accuracy of 72%.

### Typical cases presentation

**Fig. 6:**
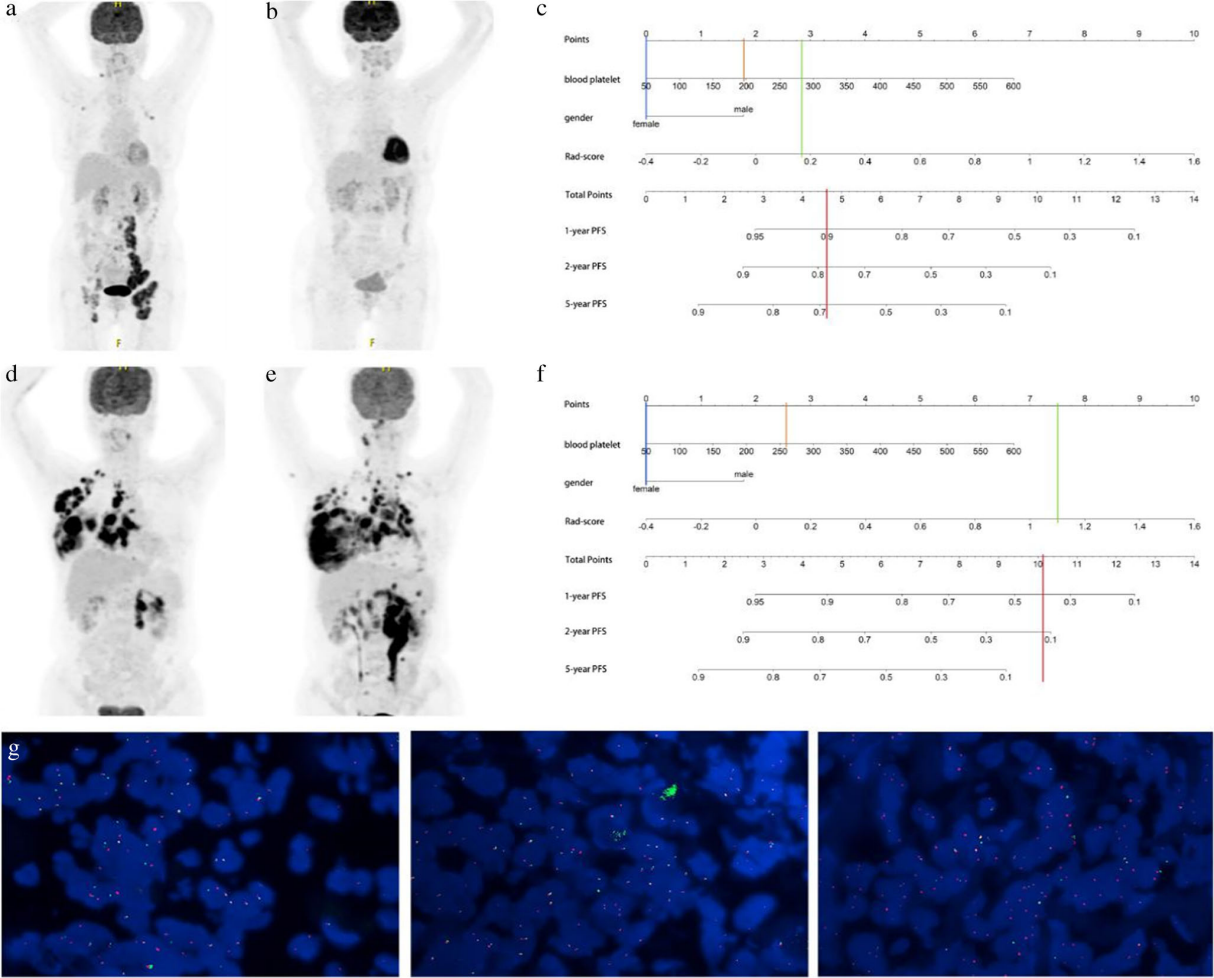
Two typical cases with DLBCL to show the clinical application of the nomogram. **a**–**c** Female, 75 years old, who underwent 6 cycles of R-CHOP regimen chemotherapy after firstly diagnosed of DLBCL (**a**) and was confirmed as completely response (CR) **b**. Blood platelet: 192, the Rad-score: 0.18. Vertical lines of each variable were drawn (**c**) and total points: (0 + 1.76 + 2.85 = 4.61). **c**–**g** Female, 46 years old, who underwent 4 cycles of R-CHOP regimen chemotherapy after firstly diagnosed of DLBCL (**d**) and was confirmed as progression disease (PD) **e**. Blood platelet: 264, the Rad-score: 1.09. Vertical lines of each variable were drawn (**f**) and total points (0 + 2.63 + 7.5 = 10.13). FISH test results confirmed as a double-hit lymphoma (**g**)

In order to demonstrate the clinical application of the radiomics nomogram, we show the maximum intensity projection images from [^18^F]FDG PET scans of several typical cases with DLBCL (Fig. 6 and Supplementary Figure 1). As the nomogram successfully predicted, the prognosis of the first case (Fig. 6a, b and c) showed no recurrence after 4.2 years of standard treatment after diagnosis. Similar to the nomogram prediction for the second case (Fig. 6d, e, f and g) that had a higher risk of recurrence, it was observed that the disease progressed 3 months after the standard treatment. It is remarkable that the immunohistochemical results of the second case showed a double-hit lymphoma (DHL), with a BCL-2 rearrangement, c-MYC rearrangement, and BCL-6 non-rearrangement (Fig. 6g).

## Discussion

In conclusion, we developed a nomogram based on Rad-score, gender, and blood platelet analyses of the cases with DLBCL for individualized prediction of recurrence, which was also validated. We validated PFS of the cases at 1, 2, and 5 years. The results demonstrated that the combined Rad-score and clinical factors model significantly improved prediction accuracy as compared to models that only included clinical factors or Rad-score alone.

The outcomes of DLBCL were conventionally evaluated only based on PFS and/or overall survival (OS). Previous studies have used 2-year PFS as an endpoint for outcomes in the disease-related DLBCL immunochemotherapy [17, 21, 22].

Therefore, in this study, we evaluated the prediction power of PFS but not the OS, especially with regard to 2-year PFS. The results of the validation and training cohort of 2-year PFS were satisfactory in this combined prediction model (training cohort: 2-year PFS AUC = 0.84, validation cohort: 2-year PFS AUC = 0.83).

Currently, SUVmax, MTV, and TLG are the most widely employed indexes in the literature for predicting survival in lymphoma patients. Some retrospective studies have noted that SUVmax may predict the histological transformation of FL [23, 24], but the GALLIUM study [25] demonstrated that SUVmax alone may provide little to no benefit.

Indeed, SUV can be affected by many factors, including but not limited to partial volume effects, the time between injection and imaging acquisition, and the decay of the injected dose [26]. Domenico et al [27] found a significant correlation between baseline MTV, TLG, and therapeutic response, which predicted outcomes (OS and PFS) of Burkitt’s lymphoma. However, these parameters can only provide information about glucose metabolism in the tumor, but not the heterogeneity of metabolism.

In our study, neither MTV nor TLG was independent predictors, and both were significantly associated with PFS, although only at the univariate level. Our results suggested that identifying tumor heterogeneity from imaging information can be a promising approach.

Radiomic features from [^18^F]FDG-PET/CT images can quantify the spatial heterogeneity of tumors and have become potential prognostic predictors of many diseases [28–31]. However, as [^18^F]FDG PET/CT radiomics has not been widely applied to predict the clinical prognosis of cancer cases, there is no consensus on the screening of texture features [28].

Nonetheless, radiomics features play an increasingly crucial role in predicting the prognosis and characterizing intratumor heterogeneity of DLBCL cases [16, 32, 33]. The high tumor heterogeneity is an essential biomarker for prognosis as it often suggests higher chances of tumor recurrence and metastasis [34]. Therefore, a radiomics approach is beneficial due to its noninvasive nature for assessing tumor heterogeneity, and can potentially improve tumor management plans for cases.

Our study found that platelet count was a significant independent predictor of progression-free survival of cases with DLBCL. The role of platelets in tumor growth and progression is very important [35]. Studies have demonstrated that high platelet counts increase the risk of metastasis [36], and are related to poorer prognosis [37–39]. A complex relationship exists between platelets and cancer pathogenesis, however. Various cancer entities can release inflammatory cytokines, which can then stimulate megakaryocyte proliferation, thereby producing platelets [40]. Laurie et al [41] concluded, from a large number of clinical and experimental studies, that within the circulatory system, platelets could assist tumor cells in evading immune elimination, promote vasculature growth arrest, and contribute to tumor growth and metastasis. Therefore, blood platelets could be a valuable biomarker for clinical cancer progression, prognosis prediction, and treatment monitoring [42].

It is noteworthy that in the prediction model, we can intuitively observe that gender does not show sufficient predictive power on the basis of univariate-associated PFS. However, the lower proportion of prediction, vis a vis PFS, does not definitively imply that gender is unimportant. In addition, Scott et al [43] have shown that the female gender is an independent positive prognostic indicator for survival. It was found that NHL cases of the female gender have a protective effect on survival. Similarly, this point of view may be further supported by findings from Jennifer et al [44] that pregnancy lowers the risk of B cell NHL. Therefore, gender cannot be excluded from the prediction model due to its clinical significance.

According to the literature [45, 46], DHL is a rapidly progressive type of DLBCL with a very poor prognosis from a pathological standpoint. Remarkably, in the second typical case (Fig. 6), DHL was confirmed by FISH test in a high-risk case with a short recurrence time period. Despite the possibility that this is due to chance, we believe that the nomogram’s prediction of high-risk cases is consistent with immunohistochemical results. Our radiomics nomogram may be used to predict and support FISH test results in future studies.

The treatment efficiency for DLBCL has increased significantly in recent years. Cases with a favorable prognosis should be given the most standard treatment to avoid adverse reactions caused by excessive treatment. For cases with relapsed or refractory DLBCL, early diagnosis and treatment are essential for effective treatment [47] (for instance, increasing the intensity of chemotherapy, using stem cell transplantation, CAR-T therapeutic options, the addition of new drugs, etc.), so that these high-risk cases can receive the best treatment timing and maximum survival benefit [48–51].

Nonetheless, there are still limitations and deficiencies in this study. First, inherent selection limitation was inevitable due to the nature of retrospective studies. Second, our data were only limited to cases from one medical center and the size was relatively small. Thus, clinical support for the prediction model is limited. Future studies with a larger external validation from multiple medical centers are required. Finally, genomic characteristics have not yet been included. Until now, radiogenomics research has primarily focused on imaging phenotypes and gene expression [52], and therefore more comprehensive studies are needed in the future.

## Conclusion

Hematological indicators are available and economically needed in the clinic. This study provides a radiomics nomogram that includes gender and blood platelet counts with a Rad-score based on [^18^F]FDG PET/CT images. Our results showed that the prediction model incorporating radiomics features is significantly more powerful than clinical indicators. This model might be used more effectively to assess the prognostic risk of pretreatment DLBCL cases and further assist clinicians in directing treatment to benefit outcomes.

## Supplementary information

ESM 1 (DOCX 277 kb)

## Abbreviations

DCA: Decision curve analysis
DHL: Double-hit lymphoma
HL: Hodgkin’s lymphoma
IL-6: Interleukin-6
IPI: International Prognostic Index
LDH: Lactate dehydrogenase
LMR: Lymphocyte-to-monocyte ratio
MIP: Maximum intensity projection
MTV: Metabolic tumor volume
NHL: Non-Hodgkin’s lymphoma
NLR: Neutrophil-to-lymphocyte ratio
OS: Overall survival
PFS: Progression-free survival
PLR: Platelet-to-lymphocyte ratio
Rad-score: Radiomics score
ROC: Receiver operating characteristic
SUV: Standardized uptake value
TLG: Total lesion glycolysis

## Declarations

### Guarantor

The scientific guarantor of this publication is Kezheng Wang.

### Statistics and biometry

One of the authors (Mingshan Li) has significant statistical expertise.

### Methodology

• retrospective

• prognostic study

• performed at one institution
