# Supplementary material for: Development and validation of a [18F]FDG PET/CT-based radiomics nomogram to predict the prognostic risk of pretreatment diffuse large B cell lymphoma patients
Source: Eur Radiol. 2022 Dec 22;33(5):3354–65. doi: 10.1007/s00330-022-09301-5 (PMC10121518; doi:10.1007/s00330-022-09301-5)
Supplement: Supplementary file 1 — (DOCX 277 kb) [file 330_2022_9301_MOESM1_ESM.docx]

**Supplementary Table 1**

| feature | cox.pValue |
| --- | --- |
| original_shape_MajorAxisLength.CT | 7.09E-09 |
| original_shape_MajorAxisLength.PET | 5.44E-08 |
| original_shape_Maximum3DDiameter.CT | 2.24E-07 |
| original_shape_Maximum3DDiameter.PET | 5.18E-07 |
| original_shape_Maximum2DDiameterRow.CT | 6.53E-07 |
| original_shape_Maximum2DDiameterColumn.CT | 8.11E-07 |
| original_shape_Elongation.CT | 1.21E-06 |
| original_shape_Elongation.PET | 5.92E-06 |
| original_shape_Maximum2DDiameterColumn.PET | 9.21E-06 |
| original_shape_Maximum2DDiameterRow.PET | 3.25E-05 |
| original_shape_Flatness.CT | 3.34E-05 |
| original_shape_Flatness.PET | 8.21E-05 |
| wavelet.HHH_ngtdm_Complexity.CT | 0.000126 |
| wavelet.HHH_firstorder_Range.CT | 0.00017 |
| wavelet.LLL_gldm_DependenceEntropy.CT | 0.0002 |
| wavelet.HHH_firstorder_Minimum.CT | 0.000271 |
| wavelet.HHH_firstorder_Maximum.CT | 0.000278 |
| original_shape_Sphericity.PET | 0.000336 |
| original_shape_MinorAxisLength.CT | 0.000358 |
| wavelet.HHH_glszm_SmallAreaHighGrayLevelEmphasis.CT | 0.000492 |
| original_glrlm_RunEntropy.CT | 0.000528 |
| wavelet.LHH_firstorder_Median.PET | 0.000529 |
| wavelet.HHH_glszm_HighGrayLevelZoneEmphasis.CT | 0.000552 |
| wavelet.HHH_glcm_Autocorrelation.CT | 0.000564 |
| wavelet.HHH_gldm_HighGrayLevelEmphasis.CT | 0.000569 |
| wavelet.HHH_glcm_JointAverage.CT | 0.00057 |
| wavelet.HHH_glcm_SumAverage.CT | 0.00057 |
| wavelet.HHH_gldm_LargeDependenceHighGrayLevelEmphasis.CT | 0.000573 |
| wavelet.HHH_glrlm_HighGrayLevelRunEmphasis.CT | 0.000578 |
| wavelet.HHH_glrlm_LongRunHighGrayLevelEmphasis.CT | 0.000593 |
| wavelet.HHH_glrlm_ShortRunHighGrayLevelEmphasis.CT | 0.000666 |
| original_glcm_ClusterShade.PET | 0.000732 |
| original_shape_Maximum2DDiameterSlice.PET | 0.000798 |
| wavelet.LLL_glrlm_RunEntropy.CT | 0.000812 |
| wavelet.HLL_glcm_Autocorrelation.PET | 0.000822 |
| wavelet.LLH_glcm_Idn.PET | 0.000841 |
| wavelet.HLL_gldm_SmallDependenceHighGrayLevelEmphasis.PET | 0.00093 |
| wavelet.HLL_gldm_HighGrayLevelEmphasis.PET | 0.000931 |
| wavelet.HLL_glrlm_ShortRunHighGrayLevelEmphasis.PET | 0.000931 |
| wavelet.HLL_glrlm_HighGrayLevelRunEmphasis.PET | 0.000931 |
| wavelet.HLL_glrlm_LongRunHighGrayLevelEmphasis.PET | 0.000931 |
| wavelet.HLL_glszm_SmallAreaHighGrayLevelEmphasis.PET | 0.000934 |
| wavelet.HLL_glszm_HighGrayLevelZoneEmphasis.PET | 0.000934 |
| wavelet.HLL_glszm_LargeAreaHighGrayLevelEmphasis.PET | 0.000951 |
| wavelet.HHH_glcm_MCC.CT | 0.000965 |
| wavelet.HLL_gldm_LargeDependenceHighGrayLevelEmphasis.PET | 0.000974 |
| original_shape_LeastAxisLength.PET | 0.001105 |
| wavelet.HHL_glszm_ZoneEntropy.CT | 0.001108 |
| wavelet.LLL_glszm_ZoneEntropy.CT | 0.00121 |
| wavelet.LLL_glcm_Idn.PET | 0.001211 |
| wavelet.LHL_glcm_SumAverage.CT | 0.001213 |
| wavelet.LHL_glcm_JointAverage.CT | 0.001213 |
| log.sigma.3.0.mm.3D_glrlm_RunLengthNonUniformity.CT | 0.001273 |
| wavelet.HHH_glcm_Idn.CT | 0.001279 |
| wavelet.LHL_firstorder_Minimum.CT | 0.001387 |
| original_glszm_ZoneEntropy.CT | 0.001653 |
| wavelet.HLL_glcm_ClusterShade.PET | 0.001728 |
| log.sigma.2.0.mm.3D_glcm_ClusterProminence.PET | 0.001789 |
| log.sigma.2.0.mm.3D_glcm_ClusterShade.PET | 0.001813 |
| wavelet.LLL_glszm_GrayLevelNonUniformity.CT | 0.001823 |
| wavelet.HLH_glcm_Idn.PET | 0.001902 |
| wavelet.HHH_glcm_Idn.PET | 0.001963 |
| original_shape_Sphericity.CT | 0.001982 |
| original_ngtdm_Coarseness.PET | 0.001991 |
| wavelet.LLL_glcm_Idmn.PET | 0.001992 |
| wavelet.LLL_glrlm_RunLengthNonUniformity.CT | 0.001996 |
| log.sigma.2.0.mm.3D_ngtdm_Coarseness.PET | 0.002006 |
| original_shape_LeastAxisLength.CT | 0.002011 |
| wavelet.HHL_glcm_ClusterShade.PET | 0.002046 |
| original_shape_MinorAxisLength.PET | 0.002048 |
| wavelet.LHH_gldm_SmallDependenceHighGrayLevelEmphasis.PET | 0.002065 |
| original_shape_SurfaceArea.CT | 0.00209 |
| wavelet.HLH_glcm_ClusterProminence.PET | 0.002098 |
| log.sigma.3.0.mm.3D_glcm_ClusterShade.PET | 0.002118 |
| wavelet.HHH_glcm_Idmn.PET | 0.002158 |
| wavelet.LLH_glcm_ClusterProminence.PET | 0.002192 |
| wavelet.HLH_glcm_ClusterShade.PET | 0.002195 |
| wavelet.HLL_glcm_ClusterProminence.PET | 0.0022 |
| wavelet.LHH_glszm_SmallAreaHighGrayLevelEmphasis.PET | 0.002213 |
| log.sigma.2.0.mm.3D_glszm_GrayLevelNonUniformity.CT | 0.002276 |
| wavelet.HHL_glcm_ClusterProminence.PET | 0.002297 |
| wavelet.HLH_glszm_HighGrayLevelZoneEmphasis.PET | 0.002332 |
| wavelet.HLH_glrlm_LongRunHighGrayLevelEmphasis.PET | 0.002335 |
| wavelet.HLH_glrlm_HighGrayLevelRunEmphasis.PET | 0.00234 |
| wavelet.HLH_glrlm_ShortRunHighGrayLevelEmphasis.PET | 0.00234 |
| wavelet.HLH_gldm_HighGrayLevelEmphasis.PET | 0.002341 |
| wavelet.HLH_glszm_SmallAreaHighGrayLevelEmphasis.PET | 0.00236 |
| original_glcm_Imc1.CT | 0.002361 |
| wavelet.HLH_gldm_SmallDependenceHighGrayLevelEmphasis.PET | 0.002365 |
| wavelet.HHH_glcm_ClusterProminence.PET | 0.002374 |
| wavelet.LHH_glrlm_ShortRunHighGrayLevelEmphasis.PET | 0.002381 |
| wavelet.LHH_glszm_HighGrayLevelZoneEmphasis.PET | 0.002391 |
| wavelet.HLL_ngtdm_Contrast.PET | 0.002391 |
| wavelet.LHH_glrlm_HighGrayLevelRunEmphasis.PET | 0.002396 |
| wavelet.LHH_gldm_HighGrayLevelEmphasis.PET | 0.002396 |
| wavelet.HHH_glszm_ZoneEntropy.CT | 0.002396 |
| wavelet.HHH_glrlm_LongRunHighGrayLevelEmphasis.PET | 0.002397 |
| wavelet.HLH_gldm_LargeDependenceHighGrayLevelEmphasis.PET | 0.002398 |
| wavelet.HHL_ngtdm_Contrast.PET | 0.002408 |
| log.sigma.2.0.mm.3D_glrlm_RunLengthNonUniformity.CT | 0.00242 |
| wavelet.LHH_glcm_ClusterProminence.PET | 0.00242 |
| wavelet.HHH_glcm_ClusterShade.PET | 0.002436 |
| wavelet.HHH_glszm_HighGrayLevelZoneEmphasis.PET | 0.002452 |
| wavelet.LHH_glrlm_LongRunHighGrayLevelEmphasis.PET | 0.002457 |
| original_glcm_Idn.PET | 0.002466 |
| wavelet.HLH_ngtdm_Coarseness.PET | 0.002477 |
| wavelet.HHH_glrlm_HighGrayLevelRunEmphasis.PET | 0.002482 |
| wavelet.HHH_gldm_HighGrayLevelEmphasis.PET | 0.002485 |
| wavelet.HLH_ngtdm_Contrast.PET | 0.002485 |
| log.sigma.2.0.mm.3D_ngtdm_Contrast.PET | 0.002488 |
| log.sigma.3.0.mm.3D_glszm_ZoneEntropy.CT | 0.002494 |
| wavelet.HHH_glrlm_ShortRunHighGrayLevelEmphasis.PET | 0.002502 |
| wavelet.LHL_ngtdm_Contrast.PET | 0.002513 |
| wavelet.HHH_glszm_SmallAreaHighGrayLevelEmphasis.PET | 0.002518 |
| wavelet.HLH_glszm_LargeAreaHighGrayLevelEmphasis.PET | 0.002528 |
| original_ngtdm_Contrast.PET | 0.002556 |
| wavelet.LHH_glcm_ClusterShade.PET | 0.002579 |
| log.sigma.3.0.mm.3D_ngtdm_Contrast.PET | 0.002617 |
| wavelet.LLL_gldm_DependenceVariance.CT | 0.002619 |
| wavelet.LLH_glcm_Idmn.PET | 0.002634 |
| wavelet.HLH_firstorder_Kurtosis.CT | 0.002649 |
| wavelet.HHL_ngtdm_Coarseness.PET | 0.002657 |
| log.sigma.3.0.mm.3D_glcm_JointEntropy.PET | 0.002663 |
| wavelet.HHH_firstorder_Kurtosis.CT | 0.002675 |
| wavelet.LHL_glcm_JointEntropy.PET | 0.002676 |
| log.sigma.2.0.mm.3D_glcm_JointEntropy.PET | 0.002682 |
| wavelet.HLL_glcm_JointEntropy.PET | 0.002724 |
| wavelet.HHH_glcm_Autocorrelation.PET | 0.002741 |
| wavelet.LLL_ngtdm_Contrast.PET | 0.00275 |
| wavelet.LHH_ngtdm_Contrast.PET | 0.002752 |
| wavelet.HHH_gldm_SmallDependenceHighGrayLevelEmphasis.PET | 0.002752 |
| wavelet.LHH_glcm_Autocorrelation.PET | 0.002758 |
| wavelet.HHH_glszm_LowGrayLevelZoneEmphasis.CT | 0.002759 |
| wavelet.HHH_ngtdm_Contrast.PET | 0.002763 |
| wavelet.HLH_glcm_DifferenceVariance.PET | 0.002918 |
| wavelet.LHL_glcm_ClusterShade.PET | 0.002976 |
| wavelet.HLH_glszm_GrayLevelVariance.PET | 0.002997 |
| wavelet.HLH_glrlm_GrayLevelVariance.PET | 0.003036 |
| wavelet.HLH_firstorder_Variance.PET | 0.003038 |
| wavelet.HLH_gldm_GrayLevelVariance.PET | 0.003039 |
| original_glrlm_RunLengthNonUniformity.CT | 0.00305 |
| wavelet.LHL_glcm_ClusterProminence.PET | 0.003059 |
| wavelet.LLH_glcm_JointEntropy.PET | 0.003069 |
| wavelet.LHH_ngtdm_Coarseness.PET | 0.003069 |
| wavelet.HLL_glszm_ZoneEntropy.CT | 0.003097 |
| wavelet.LLH_glrlm_RunLengthNonUniformity.CT | 0.003103 |
| wavelet.LHH_glcm_JointEntropy.PET | 0.003143 |
| wavelet.HLH_glcm_ClusterTendency.PET | 0.003169 |
| wavelet.HLL_glrlm_RunLengthNonUniformity.CT | 0.003172 |
| wavelet.HLH_glcm_JointEntropy.PET | 0.003176 |
| wavelet.LHH_glcm_Idn.PET | 0.003244 |
| wavelet.LLL_firstorder_Entropy.PET | 0.003281 |
| wavelet.LLH_ngtdm_Coarseness.PET | 0.003288 |
| wavelet.LLL_glrlm_RunEntropy.PET | 0.0033 |
| original_glcm_JointEntropy.PET | 0.003331 |
| log.sigma.3.0.mm.3D_ngtdm_Coarseness.PET | 0.003343 |
| log.sigma.3.0.mm.3D_glcm_ClusterProminence.PET | 0.003356 |
| wavelet.HLL_glcm_Contrast.PET | 0.003395 |
| wavelet.LLH_ngtdm_Contrast.PET | 0.003406 |
| wavelet.HHH_gldm_LargeDependenceHighGrayLevelEmphasis.PET | 0.00341 |
| wavelet.LLL_glszm_ZoneEntropy.PET | 0.003431 |
| wavelet.LLL_glcm_SumEntropy.PET | 0.003456 |
| original_glcm_ClusterProminence.PET | 0.003461 |
| wavelet.HLL_glcm_DifferenceVariance.PET | 0.003478 |
| wavelet.HHL_glcm_JointEntropy.PET | 0.003495 |
| wavelet.LLL_gldm_DependenceEntropy.PET | 0.003504 |
| wavelet.HLH_glcm_SumSquares.PET | 0.003535 |
| original_gldm_DependenceEntropy.CT | 0.003546 |
| wavelet.LHH_glcm_Idmn.PET | 0.00355 |
| wavelet.HLH_glcm_Idmn.PET | 0.003588 |
| wavelet.HLH_glcm_Autocorrelation.PET | 0.003636 |
| wavelet.HLL_glcm_SumSquares.PET | 0.003659 |
| original_glcm_Correlation.PET | 0.003668 |
| original_glcm_Imc2.CT | 0.003698 |
| wavelet.HLL_glszm_GrayLevelVariance.PET | 0.003706 |
| wavelet.HLH_glrlm_RunLengthNonUniformity.CT | 0.003707 |
| wavelet.HLL_glrlm_GrayLevelVariance.PET | 0.003716 |
| wavelet.HLL_firstorder_Variance.PET | 0.003716 |
| wavelet.HLL_gldm_GrayLevelVariance.PET | 0.003717 |
| wavelet.HHH_glcm_DifferenceVariance.PET | 0.003756 |
| wavelet.HHH_glrlm_RunLengthNonUniformity.CT | 0.003883 |
| wavelet.HLL_glcm_Idn.PET | 0.003917 |
| wavelet.HLL_glcm_ClusterTendency.PET | 0.003997 |
| wavelet.HHL_glcm_DifferenceVariance.PET | 0.004068 |
| wavelet.HHL_glrlm_RunLengthNonUniformity.CT | 0.004134 |
| wavelet.LLL_glcm_JointEntropy.PET | 0.00415 |
| wavelet.HLH_glcm_Contrast.PET | 0.004194 |
| original_glcm_Idmn.PET | 0.004225 |
| log.sigma.2.0.mm.3D_glcm_DifferenceVariance.PET | 0.004254 |
| original_glcm_SumEntropy.PET | 0.004283 |
| wavelet.LLL_glszm_SizeZoneNonUniformity.CT | 0.004364 |
| log.sigma.2.0.mm.3D_glcm_Contrast.PET | 0.004386 |
| log.sigma.2.0.mm.3D_glcm_SumSquares.PET | 0.004417 |
| wavelet.LHH_glrlm_GrayLevelNonUniformity.CT | 0.00444 |
| wavelet.LLL_glcm_Correlation.PET | 0.004482 |
| wavelet.LHL_ngtdm_Coarseness.PET | 0.004513 |
| wavelet.HHH_glrlm_GrayLevelNonUniformity.CT | 0.004657 |
| wavelet.LHH_gldm_LargeDependenceHighGrayLevelEmphasis.CT | 0.0048 |
| wavelet.LLL_gldm_DependenceNonUniformity.CT | 0.00485 |
| wavelet.LHH_gldm_GrayLevelNonUniformity.CT | 0.004852 |
| original_gldm_DependenceVariance.CT | 0.004869 |
| log.sigma.2.0.mm.3D_glszm_ZoneEntropy.CT | 0.0049 |
| log.sigma.3.0.mm.3D_glcm_Correlation.PET | 0.004903 |
| wavelet.LLL_glcm_DifferenceEntropy.PET | 0.004904 |
| wavelet.HHL_glrlm_GrayLevelNonUniformity.CT | 0.004911 |
| wavelet.LHL_glcm_SumEntropy.PET | 0.004944 |
| original_glszm_SizeZoneNonUniformity.CT | 0.004945 |
| wavelet.LLH_gldm_LargeDependenceHighGrayLevelEmphasis.PET | 0.004986 |
| log.sigma.2.0.mm.3D_glcm_Correlation.PET | 0.004996 |
| wavelet.LHH_glrlm_RunLengthNonUniformity.CT | 0.005008 |
| wavelet.LHH_glszm_LargeAreaHighGrayLevelEmphasis.PET | 0.005032 |
| wavelet.LHL_gldm_GrayLevelNonUniformity.CT | 0.005062 |
| original_shape_VoxelVolume.CT | 0.005147 |
| wavelet.LLH_glszm_LargeAreaHighGrayLevelEmphasis.PET | 0.005158 |
| wavelet.LHH_gldm_LargeDependenceHighGrayLevelEmphasis.PET | 0.005167 |
| wavelet.HLH_glrlm_GrayLevelNonUniformity.CT | 0.005187 |
| wavelet.LHL_firstorder_Range.CT | 0.005221 |
| wavelet.HHH_gldm_GrayLevelNonUniformity.CT | 0.005221 |
| original_shape_MeshVolume.CT | 0.005224 |
| wavelet.HHL_glcm_Contrast.PET | 0.005304 |
| wavelet.LLH_glrlm_LongRunHighGrayLevelEmphasis.PET | 0.005334 |
| wavelet.HHL_gldm_GrayLevelNonUniformity.CT | 0.005344 |
| wavelet.LLH_gldm_HighGrayLevelEmphasis.PET | 0.005348 |
| wavelet.LLH_glrlm_HighGrayLevelRunEmphasis.PET | 0.005349 |
| wavelet.LLH_glszm_HighGrayLevelZoneEmphasis.PET | 0.005351 |
| wavelet.LLH_glrlm_ShortRunHighGrayLevelEmphasis.PET | 0.005352 |
| original_glcm_Correlation.CT | 0.005363 |
| wavelet.LHL_glrlm_GrayLevelNonUniformity.CT | 0.005376 |
| wavelet.LLH_glszm_SmallAreaHighGrayLevelEmphasis.PET | 0.00538 |
| wavelet.LLH_gldm_SmallDependenceHighGrayLevelEmphasis.PET | 0.005418 |
| log.sigma.3.0.mm.3D_glcm_SumEntropy.PET | 0.005422 |
| log.sigma.2.0.mm.3D_glszm_GrayLevelVariance.PET | 0.005446 |
| log.sigma.3.0.mm.3D_glcm_Idn.PET | 0.005454 |
| wavelet.HLL_glcm_SumEntropy.PET | 0.005459 |
| log.sigma.2.0.mm.3D_glrlm_GrayLevelVariance.PET | 0.005465 |
| log.sigma.2.0.mm.3D_gldm_GrayLevelVariance.PET | 0.005466 |
| log.sigma.2.0.mm.3D_firstorder_Variance.PET | 0.005469 |
| wavelet.HHH_glcm_Contrast.PET | 0.005494 |
| wavelet.HHH_glcm_SumSquares.PET | 0.005518 |
| wavelet.HHH_glcm_ClusterTendency.PET | 0.005575 |
| wavelet.HHH_glszm_GrayLevelVariance.PET | 0.005585 |
| wavelet.HLH_gldm_GrayLevelNonUniformity.CT | 0.005623 |
| wavelet.LLH_glcm_SumEntropy.PET | 0.005667 |
| wavelet.HHH_glrlm_GrayLevelVariance.PET | 0.005894 |
| wavelet.HHH_gldm_GrayLevelVariance.PET | 0.005908 |
| wavelet.LLH_glcm_Autocorrelation.PET | 0.005927 |
| wavelet.HHH_firstorder_Variance.PET | 0.005937 |
| wavelet.HHL_glszm_GrayLevelVariance.PET | 0.00619 |
| wavelet.HHL_glrlm_GrayLevelVariance.PET | 0.006239 |
| wavelet.HHL_firstorder_Variance.PET | 0.006243 |
| wavelet.HHL_gldm_GrayLevelVariance.PET | 0.006244 |
| wavelet.HHH_glszm_SizeZoneNonUniformity.PET | 0.006257 |
| wavelet.LLH_glcm_JointAverage.CT | 0.006403 |
| wavelet.LLH_glcm_SumAverage.CT | 0.006403 |
| wavelet.LHL_gldm_DependenceEntropy.PET | 0.006434 |
| wavelet.HLL_glcm_Idmn.PET | 0.006472 |
| original_gldm_DependenceEntropy.PET | 0.006475 |
| wavelet.LHH_firstorder_Mean.PET | 0.006525 |
| original_shape_Maximum2DDiameterSlice.CT | 0.006657 |
| original_glszm_ZoneEntropy.PET | 0.006671 |
| wavelet.LHH_glszm_SizeZoneNonUniformity.PET | 0.006762 |
| wavelet.HLL_gldm_GrayLevelNonUniformity.CT | 0.006797 |
| wavelet.LLH_firstorder_Minimum.CT | 0.006803 |
| wavelet.HLL_glrlm_GrayLevelNonUniformity.CT | 0.006898 |
| original_glrlm_RunEntropy.PET | 0.006994 |
| wavelet.LHH_gldm_DependenceNonUniformity.CT | 0.007 |
| original_firstorder_Entropy.PET | 0.007004 |
| log.sigma.2.0.mm.3D_gldm_DependenceNonUniformity.CT | 0.007012 |
| log.sigma.3.0.mm.3D_gldm_DependenceNonUniformity.CT | 0.007045 |
| wavelet.HHL_glcm_MCC.CT | 0.007065 |
| wavelet.HHH_gldm_DependenceNonUniformity.PET | 0.007105 |
| log.sigma.2.0.mm.3D_gldm_GrayLevelNonUniformity.CT | 0.007118 |
| log.sigma.2.0.mm.3D_glcm_SumEntropy.PET | 0.007126 |
| wavelet.HHL_glcm_SumSquares.PET | 0.00714 |
| wavelet.LLH_gldm_DependenceEntropy.PET | 0.007165 |
| wavelet.LHL_glszm_ZoneEntropy.PET | 0.007221 |
| original_firstorder_90Percentile.CT | 0.007307 |
| log.sigma.3.0.mm.3D_glcm_DifferenceVariance.PET | 0.007317 |
| wavelet.LHH_glcm_Idn.CT | 0.007351 |
| wavelet.LHH_glrlm_LongRunHighGrayLevelEmphasis.CT | 0.007361 |
| log.sigma.3.0.mm.3D_glrlm_GrayLevelNonUniformity.CT | 0.007391 |
| log.sigma.2.0.mm.3D_glcm_Idn.PET | 0.007472 |
| wavelet.HHL_glcm_Idn.CT | 0.007481 |
| wavelet.LHL_glrlm_RunLengthNonUniformity.CT | 0.007516 |
| wavelet.HLL_gldm_DependenceEntropy.PET | 0.00753 |
| log.sigma.3.0.mm.3D_gldm_DependenceEntropy.PET | 0.007532 |
| original_firstorder_TotalEnergy.CT | 0.00756 |
| original_firstorder_Energy.CT | 0.007566 |
| wavelet.LHH_gldm_DependenceNonUniformity.PET | 0.007645 |
| wavelet.LHL_glcm_Contrast.PET | 0.007765 |
| wavelet.HHL_glszm_SizeZoneNonUniformity.PET | 0.007781 |
| wavelet.LHL_glrlm_ShortRunHighGrayLevelEmphasis.CT | 0.00794 |
| wavelet.LLH_glszm_ZoneEntropy.PET | 0.007957 |
| original_shape_SurfaceArea.PET | 0.008295 |
| log.sigma.3.0.mm.3D_glcm_Contrast.PET | 0.008305 |
| log.sigma.2.0.mm.3D_firstorder_TotalEnergy.CT | 0.008332 |
| log.sigma.2.0.mm.3D_firstorder_Energy.CT | 0.008336 |
| wavelet.HLL_glszm_ZoneEntropy.PET | 0.008431 |
| wavelet.HHH_gldm_DependenceNonUniformity.CT | 0.00844 |
| wavelet.LHL_glrlm_RunEntropy.PET | 0.00852 |
| wavelet.HHL_gldm_DependenceNonUniformity.CT | 0.008573 |
| wavelet.HLH_firstorder_Minimum.PET | 0.008632 |
| wavelet.HHH_ngtdm_Coarseness.PET | 0.008662 |
| wavelet.LHL_firstorder_Entropy.PET | 0.008711 |
| log.sigma.3.0.mm.3D_glszm_ZoneEntropy.PET | 0.008723 |
| wavelet.LLH_gldm_DependenceNonUniformity.CT | 0.008762 |
| wavelet.LHL_gldm_DependenceNonUniformity.CT | 0.008879 |
| wavelet.LLH_glrlm_RunEntropy.PET | 0.008987 |
| wavelet.HHL_gldm_DependenceNonUniformity.PET | 0.00901 |
| wavelet.HLH_gldm_DependenceNonUniformity.CT | 0.009015 |
| wavelet.LLH_glcm_ClusterShade.PET | 0.009066 |
| log.sigma.2.0.mm.3D_glrlm_GrayLevelNonUniformity.CT | 0.009096 |
| wavelet.LLH_firstorder_Entropy.PET | 0.009119 |
| log.sigma.3.0.mm.3D_glszm_GrayLevelNonUniformity.CT | 0.009347 |
| wavelet.HLH_glcm_JointAverage.PET | 0.009372 |
| wavelet.HLH_glcm_SumAverage.PET | 0.009372 |
| wavelet.LHH_glcm_Contrast.PET | 0.009411 |
| wavelet.HLH_glszm_SizeZoneNonUniformity.PET | 0.009539 |
| log.sigma.3.0.mm.3D_gldm_GrayLevelNonUniformity.CT | 0.00964 |
| wavelet.LLL_firstorder_Energy.CT | 0.009694 |
| wavelet.LLL_firstorder_TotalEnergy.CT | 0.009734 |
| wavelet.HLL_glrlm_RunEntropy.PET | 0.009752 |
| wavelet.LHL_glszm_SizeZoneNonUniformity.CT | 0.009794 |
| log.sigma.2.0.mm.3D_gldm_DependenceEntropy.PET | 0.009866 |
| log.sigma.3.0.mm.3D_firstorder_TotalEnergy.CT | 0.00987 |
| log.sigma.3.0.mm.3D_firstorder_Energy.CT | 0.009876 |
| wavelet.HLL_firstorder_Entropy.PET | 0.009922 |
| wavelet.HLH_firstorder_Maximum.CT | 0.009933 |
| wavelet.LLH_glrlm_GrayLevelNonUniformity.CT | 0.010065 |
| wavelet.LLL_glcm_Imc2.CT | 0.010074 |
| wavelet.HLL_glszm_SizeZoneNonUniformity.CT | 0.0101 |
| wavelet.HLH_firstorder_Range.PET | 0.010124 |
| wavelet.LLH_firstorder_Kurtosis.PET | 0.010156 |
| wavelet.HLH_glcm_Imc1.PET | 0.010241 |
| log.sigma.2.0.mm.3D_firstorder_TotalEnergy.PET | 0.010324 |
| log.sigma.2.0.mm.3D_firstorder_Energy.PET | 0.010393 |
| original_glrlm_GrayLevelNonUniformityNormalized.CT | 0.010477 |
| wavelet.LLH_gldm_GrayLevelNonUniformity.CT | 0.010499 |
| wavelet.HLL_glcm_Idn.CT | 0.010506 |
| wavelet.HLL_gldm_DependenceNonUniformity.CT | 0.01057 |
| wavelet.LHH_glcm_Autocorrelation.CT | 0.01063 |
| wavelet.HHH_gldm_SmallDependenceLowGrayLevelEmphasis.CT | 0.010702 |
| log.sigma.2.0.mm.3D_glcm_ClusterTendency.PET | 0.010711 |
| log.sigma.3.0.mm.3D_glrlm_RunEntropy.CT | 0.010731 |
| wavelet.HHL_firstorder_Maximum.CT | 0.010855 |
| log.sigma.3.0.mm.3D_glrlm_RunEntropy.PET | 0.010859 |
| wavelet.HHH_glcm_Idmn.CT | 0.010983 |
| wavelet.LHH_glcm_SumAverage.CT | 0.011005 |
| wavelet.LHH_glcm_JointAverage.CT | 0.011005 |
| log.sigma.3.0.mm.3D_firstorder_Energy.PET | 0.01104 |
| wavelet.HLH_gldm_DependenceNonUniformity.PET | 0.011066 |
| wavelet.LHH_firstorder_Kurtosis.CT | 0.0111 |
| log.sigma.3.0.mm.3D_firstorder_TotalEnergy.PET | 0.0111 |
| wavelet.LHH_gldm_HighGrayLevelEmphasis.CT | 0.011104 |
| wavelet.LHH_glcm_Imc1.PET | 0.011151 |
| log.sigma.3.0.mm.3D_firstorder_Entropy.PET | 0.01118 |
| original_glcm_Contrast.PET | 0.011211 |
| wavelet.HHL_glcm_ClusterTendency.PET | 0.011229 |
| original_glcm_DifferenceEntropy.PET | 0.011243 |
| wavelet.LHL_glrlm_HighGrayLevelRunEmphasis.CT | 0.011255 |
| wavelet.LHL_glcm_Autocorrelation.CT | 0.011309 |
| log.sigma.3.0.mm.3D_glcm_Idmn.PET | 0.011363 |
| wavelet.LHL_gldm_HighGrayLevelEmphasis.CT | 0.01138 |
| wavelet.LHH_glrlm_HighGrayLevelRunEmphasis.CT | 0.011427 |
| wavelet.HHL_glszm_GrayLevelNonUniformity.CT | 0.011462 |
| wavelet.LLH_glcm_Autocorrelation.CT | 0.011602 |
| original_glcm_DifferenceVariance.PET | 0.011784 |
| wavelet.LHL_gldm_DependenceNonUniformity.PET | 0.011859 |
| wavelet.LLH_gldm_HighGrayLevelEmphasis.CT | 0.012114 |
| log.sigma.2.0.mm.3D_glszm_ZoneEntropy.PET | 0.012142 |
| wavelet.LHH_glcm_JointAverage.PET | 0.012231 |
| wavelet.LHH_glcm_SumAverage.PET | 0.012231 |
| log.sigma.2.0.mm.3D_glszm_SizeZoneNonUniformity.CT | 0.012232 |
| wavelet.LHH_glszm_HighGrayLevelZoneEmphasis.CT | 0.012245 |
| wavelet.LHL_glszm_SizeZoneNonUniformity.PET | 0.012289 |
| wavelet.HLH_glcm_Idn.CT | 0.012324 |
| wavelet.LLH_glrlm_HighGrayLevelRunEmphasis.CT | 0.01244 |
| wavelet.LHL_glszm_HighGrayLevelZoneEmphasis.CT | 0.012495 |
| log.sigma.2.0.mm.3D_glszm_SizeZoneNonUniformity.PET | 0.012554 |
| wavelet.LHH_glszm_GrayLevelVariance.PET | 0.012587 |
| wavelet.LLH_gldm_LargeDependenceHighGrayLevelEmphasis.CT | 0.01264 |
| wavelet.LHH_firstorder_Minimum.CT | 0.012701 |
| log.sigma.3.0.mm.3D_glszm_SizeZoneNonUniformity.PET | 0.012763 |
| log.sigma.2.0.mm.3D_glcm_Idmn.PET | 0.012805 |
| wavelet.LHH_glrlm_GrayLevelVariance.PET | 0.012828 |
| wavelet.LHH_gldm_GrayLevelVariance.PET | 0.012843 |
| wavelet.LHH_firstorder_Variance.PET | 0.012856 |
| log.sigma.2.0.mm.3D_gldm_DependenceNonUniformity.PET | 0.012933 |
| log.sigma.3.0.mm.3D_gldm_DependenceNonUniformity.PET | 0.013004 |
| wavelet.HLH_firstorder_Maximum.PET | 0.013206 |
| original_glszm_GrayLevelNonUniformity.CT | 0.013259 |
| wavelet.HLL_glcm_JointAverage.PET | 0.013269 |
| wavelet.HLL_glcm_SumAverage.PET | 0.013269 |
| wavelet.HHH_firstorder_TotalEnergy.CT | 0.01328 |
| wavelet.HHH_firstorder_Energy.CT | 0.013409 |
| wavelet.LLH_glszm_GrayLevelNonUniformity.CT | 0.01358 |
| wavelet.LLH_glcm_JointAverage.PET | 0.013839 |
| wavelet.LLH_glcm_SumAverage.PET | 0.013839 |
| wavelet.LHH_gldm_DependenceEntropy.PET | 0.013887 |
| wavelet.HHH_glszm_ZoneEntropy.PET | 0.013904 |
| wavelet.LHH_firstorder_TotalEnergy.PET | 0.014011 |
| wavelet.LLL_firstorder_90Percentile.CT | 0.014013 |
| wavelet.LHH_firstorder_Energy.PET | 0.014038 |
| original_gldm_DependenceNonUniformity.CT | 0.014243 |
| wavelet.HLL_firstorder_TotalEnergy.CT | 0.014265 |
| wavelet.HLL_firstorder_Energy.CT | 0.014287 |
| wavelet.HHL_glcm_Imc1.PET | 0.014304 |
| wavelet.LHL_glcm_DifferenceEntropy.PET | 0.014485 |
| wavelet.HLL_gldm_DependenceNonUniformity.PET | 0.014583 |
| wavelet.HLL_ngtdm_Coarseness.PET | 0.014636 |
| wavelet.HHH_glcm_Imc1.PET | 0.014696 |
| wavelet.HLH_gldm_DependenceEntropy.PET | 0.014827 |
| wavelet.LLH_glcm_DifferenceEntropy.PET | 0.014836 |
| wavelet.HHH_glszm_GrayLevelNonUniformityNormalized.CT | 0.014874 |
| wavelet.HHH_glcm_JointEntropy.PET | 0.014895 |
| wavelet.HLL_glszm_GrayLevelNonUniformity.CT | 0.014935 |
| wavelet.HLL_glszm_SizeZoneNonUniformity.PET | 0.014936 |
| wavelet.LHH_glcm_DifferenceVariance.PET | 0.014975 |
| wavelet.LLL_ngtdm_Coarseness.PET | 0.014995 |
| wavelet.LHH_glcm_SumSquares.PET | 0.015071 |
| wavelet.LLH_glrlm_ShortRunHighGrayLevelEmphasis.CT | 0.015156 |
| wavelet.LLL_glcm_Correlation.CT | 0.015235 |
| wavelet.LLH_glszm_HighGrayLevelZoneEmphasis.CT | 0.015278 |
| wavelet.HHH_glrlm_ShortRunLowGrayLevelEmphasis.CT | 0.015368 |
| wavelet.HLH_glszm_ZoneEntropy.CT | 0.015457 |
| wavelet.HHH_glszm_GrayLevelNonUniformity.CT | 0.015596 |
| original_glrlm_GrayLevelNonUniformity.CT | 0.015599 |
| wavelet.HLL_glszm_SmallAreaLowGrayLevelEmphasis.CT | 0.015659 |
| log.sigma.3.0.mm.3D_glcm_MCC.CT | 0.015689 |
| original_glcm_Idn.CT | 0.01576 |
| wavelet.LHH_firstorder_Range.CT | 0.015838 |
| wavelet.LLH_firstorder_Minimum.PET | 0.015894 |
| wavelet.LHL_glszm_ZoneEntropy.CT | 0.015942 |
| wavelet.HLH_firstorder_TotalEnergy.CT | 0.016458 |
| wavelet.LHL_glcm_Idn.PET | 0.016474 |
| wavelet.HLH_firstorder_Energy.CT | 0.016513 |
| wavelet.HLL_glcm_DifferenceEntropy.PET | 0.016657 |
| log.sigma.3.0.mm.3D_glcm_Correlation.CT | 0.016686 |
| wavelet.HHL_glcm_MCC.PET | 0.016991 |
| wavelet.HHH_glrlm_LowGrayLevelRunEmphasis.CT | 0.017097 |
| log.sigma.3.0.mm.3D_glcm_SumSquares.PET | 0.017125 |
| original_gldm_DependenceNonUniformity.PET | 0.017162 |
| wavelet.HHH_gldm_LowGrayLevelEmphasis.CT | 0.017336 |
| wavelet.HLL_firstorder_Kurtosis.CT | 0.017381 |
| wavelet.HLH_glcm_MCC.CT | 0.0174 |
| wavelet.LHL_glcm_Idmn.PET | 0.017584 |
| log.sigma.2.0.mm.3D_glrlm_RunEntropy.PET | 0.017649 |
| wavelet.HLL_firstorder_Minimum.PET | 0.017943 |
| wavelet.LLH_firstorder_Range.PET | 0.018044 |
| wavelet.HLH_glszm_GrayLevelNonUniformity.CT | 0.018071 |
| wavelet.HHL_gldm_DependenceEntropy.PET | 0.018072 |
| wavelet.HHH_glszm_SmallAreaLowGrayLevelEmphasis.CT | 0.018209 |
| wavelet.LLH_gldm_DependenceNonUniformity.PET | 0.0184 |
| wavelet.LHL_glcm_Idn.CT | 0.01844 |
| wavelet.HLH_firstorder_Range.CT | 0.018522 |
| log.sigma.2.0.mm.3D_firstorder_Entropy.PET | 0.018536 |
| original_glszm_SizeZoneNonUniformity.PET | 0.018743 |
| wavelet.HHL_firstorder_TotalEnergy.CT | 0.018874 |
| wavelet.HHL_firstorder_Energy.CT | 0.019011 |
| wavelet.LHH_glrlm_ShortRunHighGrayLevelEmphasis.CT | 0.019424 |
| wavelet.LHH_glszm_SmallAreaHighGrayLevelEmphasis.CT | 0.019534 |
| wavelet.HHH_glrlm_RunLengthNonUniformity.PET | 0.019682 |
| wavelet.LLH_glszm_ZoneEntropy.CT | 0.020027 |
| wavelet.LLH_glszm_SizeZoneNonUniformity.PET | 0.020058 |
| wavelet.HLL_glszm_LowGrayLevelZoneEmphasis.CT | 0.020182 |
| wavelet.LHH_firstorder_Range.PET | 0.020292 |
| wavelet.HHL_glcm_Idmn.CT | 0.020696 |
| wavelet.HHH_firstorder_Minimum.PET | 0.020771 |
| wavelet.HLL_firstorder_Minimum.CT | 0.02081 |
| log.sigma.2.0.mm.3D_glcm_Imc1.PET | 0.020846 |
| wavelet.LHH_firstorder_Minimum.PET | 0.021069 |
| wavelet.HHH_glcm_SumAverage.PET | 0.021243 |
| wavelet.HHH_glcm_JointAverage.PET | 0.021243 |
| wavelet.HHH_glrlm_RunVariance.CT | 0.021312 |
| wavelet.HHH_gldm_LargeDependenceEmphasis.CT | 0.021338 |
| wavelet.HLL_glcm_JointAverage.CT | 0.021402 |
| wavelet.HLL_glcm_SumAverage.CT | 0.021402 |
| wavelet.LHL_ngtdm_Complexity.CT | 0.021418 |
| wavelet.LHL_firstorder_Median.PET | 0.021433 |
| wavelet.HLH_glcm_Correlation.PET | 0.021502 |
| wavelet.LHH_gldm_SmallDependenceLowGrayLevelEmphasis.CT | 0.021567 |
| wavelet.HHH_gldm_DependenceEntropy.PET | 0.021733 |
| wavelet.HHL_glrlm_RunEntropy.CT | 0.021765 |
| wavelet.LLH_glszm_SizeZoneNonUniformity.CT | 0.021823 |
| wavelet.LHH_glcm_MCC.PET | 0.021847 |
| original_gldm_GrayLevelNonUniformity.CT | 0.021896 |
| wavelet.HLL_firstorder_Range.PET | 0.02208 |
| wavelet.LHH_glszm_ZoneEntropy.PET | 0.022111 |
| wavelet.HHH_gldm_SmallDependenceHighGrayLevelEmphasis.CT | 0.022204 |
| wavelet.LLH_firstorder_Energy.PET | 0.022477 |
| wavelet.HLL_glrlm_LongRunLowGrayLevelEmphasis.CT | 0.022604 |
| wavelet.LLH_firstorder_TotalEnergy.PET | 0.022616 |
| wavelet.LHH_gldm_LargeDependenceEmphasis.CT | 0.022693 |
| wavelet.LHH_gldm_LargeDependenceLowGrayLevelEmphasis.PET | 0.022711 |
| wavelet.HHH_firstorder_Range.PET | 0.022734 |
| wavelet.HHL_glcm_Idn.PET | 0.022917 |
| wavelet.HLH_glcm_SumEntropy.PET | 0.022987 |
| wavelet.HHH_glrlm_RunPercentage.CT | 0.022994 |
| wavelet.HLH_glszm_ZoneEntropy.PET | 0.023082 |
| wavelet.LLH_glrlm_LongRunHighGrayLevelEmphasis.CT | 0.023199 |
| wavelet.LLL_glrlm_GrayLevelNonUniformity.CT | 0.023567 |
| wavelet.LHL_gldm_LargeDependenceHighGrayLevelEmphasis.CT | 0.023642 |
| wavelet.LLH_glcm_Contrast.PET | 0.023647 |
| wavelet.HLH_glszm_LargeAreaLowGrayLevelEmphasis.PET | 0.023712 |
| wavelet.LLL_ngtdm_Coarseness.CT | 0.023986 |
| wavelet.LHH_glcm_SumEntropy.PET | 0.024051 |
| wavelet.HLL_glszm_LargeAreaHighGrayLevelEmphasis.CT | 0.024194 |
| wavelet.HLL_gldm_LargeDependenceLowGrayLevelEmphasis.CT | 0.024237 |
| original_shape_SurfaceVolumeRatio.CT | 0.024278 |
| wavelet.LHL_firstorder_TotalEnergy.CT | 0.024293 |
| wavelet.LHL_firstorder_Energy.CT | 0.024348 |
| wavelet.LLL_glcm_Idn.CT | 0.024557 |
| original_ngtdm_Coarseness.CT | 0.024593 |
| wavelet.LHH_firstorder_Maximum.PET | 0.024621 |
| wavelet.HHH_glrlm_RunEntropy.CT | 0.024838 |
| wavelet.HHL_ngtdm_Contrast.CT | 0.025301 |
| log.sigma.2.0.mm.3D_glcm_SumAverage.CT | 0.025853 |
| log.sigma.2.0.mm.3D_glcm_JointAverage.CT | 0.025853 |
| log.sigma.2.0.mm.3D_firstorder_Minimum.CT | 0.025862 |
| wavelet.LLH_firstorder_TotalEnergy.CT | 0.025882 |
| wavelet.LLH_firstorder_Energy.CT | 0.025902 |
| wavelet.HLL_firstorder_TotalEnergy.PET | 0.025989 |
| wavelet.HLL_firstorder_Energy.PET | 0.026093 |
| wavelet.LLH_firstorder_Kurtosis.CT | 0.026101 |
| wavelet.HHL_glcm_Idmn.PET | 0.026152 |
| log.sigma.2.0.mm.3D_glrlm_ShortRunLowGrayLevelEmphasis.CT | 0.026214 |
| original_glcm_SumSquares.PET | 0.026225 |
| wavelet.HLL_ngtdm_Coarseness.CT | 0.026286 |
| wavelet.HHH_glszm_GrayLevelVariance.CT | 0.026296 |
| wavelet.HHL_firstorder_Range.CT | 0.026615 |
| wavelet.LLH_glcm_Idn.CT | 0.02667 |
| wavelet.LHH_firstorder_Mean.CT | 0.026676 |
| wavelet.LLH_gldm_SmallDependenceLowGrayLevelEmphasis.CT | 0.026714 |
| original_firstorder_Range.PET | 0.026731 |
| wavelet.HLH_glszm_SizeZoneNonUniformity.CT | 0.026775 |
| wavelet.HHH_glrlm_LongRunEmphasis.CT | 0.026829 |
| original_firstorder_Maximum.PET | 0.026881 |
| log.sigma.3.0.mm.3D_ngtdm_Coarseness.CT | 0.026921 |
| wavelet.LLH_glszm_SmallAreaLowGrayLevelEmphasis.CT | 0.026954 |
| wavelet.LHH_firstorder_Maximum.CT | 0.026954 |
| original_firstorder_Minimum.PET | 0.027164 |
| log.sigma.2.0.mm.3D_glrlm_RunEntropy.CT | 0.027167 |
| wavelet.HHH_ngtdm_Coarseness.CT | 0.027396 |
| wavelet.LLL_gldm_DependenceNonUniformity.PET | 0.027452 |
| wavelet.LLH_ngtdm_Coarseness.CT | 0.02759 |
| log.sigma.2.0.mm.3D_ngtdm_Coarseness.CT | 0.027665 |
| wavelet.LHL_ngtdm_Coarseness.CT | 0.027813 |
| wavelet.HLH_ngtdm_Coarseness.CT | 0.027871 |
| wavelet.HHL_glcm_SumEntropy.PET | 0.028017 |
| wavelet.LHL_glcm_SumSquares.PET | 0.028067 |
| wavelet.HHL_glszm_ZoneEntropy.PET | 0.028121 |
| wavelet.HLH_firstorder_TotalEnergy.PET | 0.028131 |
| wavelet.HLH_firstorder_Energy.PET | 0.028195 |
| wavelet.LLL_firstorder_Range.PET | 0.02831 |
| wavelet.LHH_glrlm_RunPercentage.CT | 0.028356 |
| wavelet.LHH_glcm_ClusterTendency.PET | 0.028468 |
| wavelet.HHH_glcm_MCC.PET | 0.028547 |
| wavelet.HHH_glszm_LowGrayLevelZoneEmphasis.PET | 0.028733 |
| wavelet.LHL_firstorder_TotalEnergy.PET | 0.028966 |
| wavelet.LHL_firstorder_Energy.PET | 0.029117 |
| wavelet.LLH_glcm_Imc1.PET | 0.029178 |
| wavelet.LLH_firstorder_Skewness.CT | 0.029326 |
| wavelet.LLH_glszm_SmallAreaHighGrayLevelEmphasis.CT | 0.029414 |
| wavelet.HHL_ngtdm_Coarseness.CT | 0.029415 |
| wavelet.LHL_gldm_SmallDependenceHighGrayLevelEmphasis.CT | 0.029537 |
| log.sigma.3.0.mm.3D_glcm_DifferenceEntropy.PET | 0.029591 |
| wavelet.HHL_glrlm_RunVariance.CT | 0.029613 |
| wavelet.HHH_glrlm_LongRunLowGrayLevelEmphasis.PET | 0.029671 |
| wavelet.HHH_glszm_GrayLevelNonUniformity.PET | 0.029755 |
| wavelet.HHL_gldm_LargeDependenceEmphasis.CT | 0.029927 |
| wavelet.HHH_glszm_SmallAreaLowGrayLevelEmphasis.PET | 0.030183 |
| log.sigma.2.0.mm.3D_glszm_SmallAreaLowGrayLevelEmphasis.CT | 0.030246 |
| wavelet.HLH_glrlm_RunVariance.CT | 0.030367 |
| log.sigma.3.0.mm.3D_glszm_SizeZoneNonUniformity.CT | 0.030382 |
| log.sigma.3.0.mm.3D_glcm_Imc1.PET | 0.030479 |
| wavelet.LHH_ngtdm_Coarseness.CT | 0.030932 |
| original_glszm_SmallAreaLowGrayLevelEmphasis.CT | 0.031003 |
| wavelet.HHH_glszm_SizeZoneNonUniformity.CT | 0.031106 |
| wavelet.LLH_firstorder_Maximum.PET | 0.031133 |
| original_glcm_MCC.CT | 0.031147 |
| wavelet.HLL_glrlm_LowGrayLevelRunEmphasis.CT | 0.031163 |
| wavelet.LLL_glszm_SizeZoneNonUniformity.PET | 0.031186 |
| wavelet.HLL_gldm_LowGrayLevelEmphasis.CT | 0.03125 |
| wavelet.HLH_gldm_SmallDependenceLowGrayLevelEmphasis.CT | 0.031361 |
| wavelet.LHH_glcm_Idmn.CT | 0.031759 |
| wavelet.HHH_gldm_SmallDependenceLowGrayLevelEmphasis.PET | 0.031808 |
| wavelet.HHH_glrlm_RunLengthNonUniformityNormalized.CT | 0.031919 |
| log.sigma.3.0.mm.3D_glcm_JointAverage.CT | 0.03201 |
| log.sigma.3.0.mm.3D_glcm_SumAverage.CT | 0.03201 |
| wavelet.HLL_glrlm_ShortRunLowGrayLevelEmphasis.CT | 0.032256 |
| wavelet.HLL_glcm_Imc1.PET | 0.032303 |
| wavelet.HHH_glrlm_LowGrayLevelRunEmphasis.PET | 0.032398 |
| log.sigma.3.0.mm.3D_firstorder_Minimum.CT | 0.03252 |
| wavelet.HLH_gldm_LargeDependenceLowGrayLevelEmphasis.PET | 0.032586 |
| wavelet.LHH_glszm_LargeAreaLowGrayLevelEmphasis.PET | 0.032727 |
| wavelet.HHH_glrlm_ShortRunLowGrayLevelEmphasis.PET | 0.032812 |
| wavelet.HLH_glcm_Idmn.CT | 0.032945 |
| wavelet.HHH_gldm_LowGrayLevelEmphasis.PET | 0.032985 |
| wavelet.HLL_glcm_Idmn.CT | 0.033224 |
| wavelet.LHL_glrlm_LongRunHighGrayLevelEmphasis.CT | 0.033229 |
| log.sigma.2.0.mm.3D_glrlm_LowGrayLevelRunEmphasis.CT | 0.033249 |
| wavelet.HHH_glrlm_ShortRunEmphasis.CT | 0.033338 |
| wavelet.HHH_firstorder_Energy.PET | 0.033383 |
| wavelet.HHH_firstorder_TotalEnergy.PET | 0.033383 |
| wavelet.HLL_firstorder_Kurtosis.PET | 0.033486 |
| log.sigma.2.0.mm.3D_glcm_MCC.CT | 0.03352 |
| log.sigma.2.0.mm.3D_gldm_LargeDependenceLowGrayLevelEmphasis.CT | 0.033589 |
| log.sigma.2.0.mm.3D_glszm_LowGrayLevelZoneEmphasis.CT | 0.033806 |
| wavelet.LHL_glszm_SmallAreaHighGrayLevelEmphasis.CT | 0.033994 |
| original_firstorder_Range.CT | 0.034501 |
| log.sigma.2.0.mm.3D_gldm_LowGrayLevelEmphasis.CT | 0.034653 |
| wavelet.HHL_glrlm_RunPercentage.CT | 0.034786 |
| wavelet.HLH_glrlm_LongRunLowGrayLevelEmphasis.PET | 0.035123 |
| wavelet.LHH_glszm_LargeAreaHighGrayLevelEmphasis.CT | 0.035307 |
| wavelet.LHL_firstorder_Maximum.PET | 0.035329 |
| wavelet.HHL_glrlm_RunLengthNonUniformity.PET | 0.035329 |
| wavelet.HHL_firstorder_Median.PET | 0.035683 |
| wavelet.HLH_glrlm_LongRunEmphasis.CT | 0.035944 |
| log.sigma.3.0.mm.3D_gldm_GrayLevelVariance.PET | 0.036041 |
| log.sigma.3.0.mm.3D_firstorder_Variance.PET | 0.036059 |
| log.sigma.3.0.mm.3D_glrlm_GrayLevelVariance.PET | 0.036092 |
| original_glszm_LowGrayLevelZoneEmphasis.CT | 0.036107 |
| wavelet.HLH_ngtdm_Contrast.CT | 0.036425 |
| wavelet.HLH_gldm_LowGrayLevelEmphasis.PET | 0.036532 |
| log.sigma.3.0.mm.3D_glszm_GrayLevelVariance.PET | 0.036715 |
| wavelet.LLL_firstorder_Maximum.PET | 0.036765 |
| wavelet.LHH_glrlm_LongRunLowGrayLevelEmphasis.PET | 0.03678 |
| wavelet.LHH_glrlm_RunLengthNonUniformity.PET | 0.036889 |
| wavelet.HLH_glrlm_LowGrayLevelRunEmphasis.PET | 0.036945 |
| wavelet.LLL_glszm_LowGrayLevelZoneEmphasis.CT | 0.037022 |
| wavelet.LHH_firstorder_TotalEnergy.CT | 0.037248 |
| wavelet.LLL_firstorder_Range.CT | 0.037324 |
| wavelet.LHH_firstorder_Energy.CT | 0.037381 |
| wavelet.HLH_glrlm_ShortRunLowGrayLevelEmphasis.PET | 0.037475 |
| wavelet.LHH_glrlm_ShortRunLowGrayLevelEmphasis.CT | 0.037526 |
| log.sigma.2.0.mm.3D_glcm_DifferenceEntropy.PET | 0.037777 |
| wavelet.LHH_gldm_LowGrayLevelEmphasis.PET | 0.037817 |
| wavelet.LHH_glrlm_LowGrayLevelRunEmphasis.PET | 0.037852 |
| wavelet.LHH_glrlm_ShortRunLowGrayLevelEmphasis.PET | 0.038099 |
| wavelet.LHH_ngtdm_Contrast.CT | 0.038117 |
| wavelet.LHH_glszm_LowGrayLevelZoneEmphasis.PET | 0.0386 |
| wavelet.LLH_glszm_LowGrayLevelZoneEmphasis.CT | 0.038643 |
| wavelet.LLL_glrlm_ShortRunLowGrayLevelEmphasis.CT | 0.038715 |
| wavelet.LLH_glcm_Idmn.CT | 0.03878 |
| wavelet.HLH_gldm_LargeDependenceEmphasis.CT | 0.038821 |
| original_glcm_Idmn.CT | 0.038921 |
| wavelet.HLL_ngtdm_Contrast.CT | 0.038922 |
| wavelet.LHL_glcm_DifferenceVariance.PET | 0.038948 |
| wavelet.LHL_gldm_LargeDependenceEmphasis.CT | 0.039813 |
| wavelet.HHL_glrlm_LongRunEmphasis.CT | 0.03993 |
| wavelet.HHL_firstorder_Energy.PET | 0.040043 |
| wavelet.HHH_gldm_LargeDependenceLowGrayLevelEmphasis.CT | 0.040114 |
| wavelet.HHL_firstorder_TotalEnergy.PET | 0.040221 |
| log.sigma.3.0.mm.3D_firstorder_Uniformity.PET | 0.040311 |
| log.sigma.3.0.mm.3D_glrlm_GrayLevelNonUniformityNormalized.PET | 0.040375 |
| wavelet.HLH_glrlm_RunLengthNonUniformity.PET | 0.040403 |
| wavelet.LHH_glszm_SmallAreaLowGrayLevelEmphasis.PET | 0.040572 |
| wavelet.HHH_glrlm_LongRunLowGrayLevelEmphasis.CT | 0.040748 |
| log.sigma.2.0.mm.3D_glrlm_LongRunLowGrayLevelEmphasis.CT | 0.040792 |
| wavelet.HHH_firstorder_Maximum.PET | 0.040801 |
| log.sigma.3.0.mm.3D_glszm_GrayLevelNonUniformityNormalized.PET | 0.041224 |
| original_gldm_SmallDependenceLowGrayLevelEmphasis.CT | 0.041468 |
| wavelet.HHL_glszm_GrayLevelNonUniformityNormalized.CT | 0.041523 |
| wavelet.HLL_glcm_ClusterShade.CT | 0.041795 |
| log.sigma.3.0.mm.3D_glcm_Idn.CT | 0.042121 |
| wavelet.HHH_glcm_MaximumProbability.PET | 0.042167 |
| wavelet.HLL_firstorder_Maximum.PET | 0.042196 |
| wavelet.HLL_firstorder_Range.CT | 0.042302 |
| log.sigma.3.0.mm.3D_glcm_Idmn.CT | 0.042473 |
| original_gldm_SmallDependenceLowGrayLevelEmphasis.PET | 0.042625 |
| wavelet.LHH_gldm_SmallDependenceLowGrayLevelEmphasis.PET | 0.04264 |
| original_glszm_SmallAreaLowGrayLevelEmphasis.PET | 0.04269 |
| wavelet.LHL_gldm_SmallDependenceLowGrayLevelEmphasis.CT | 0.042932 |
| wavelet.HLH_gldm_LargeDependenceHighGrayLevelEmphasis.CT | 0.042939 |
| original_glszm_GrayLevelNonUniformityNormalized.CT | 0.043261 |
| original_glszm_LowGrayLevelZoneEmphasis.PET | 0.043547 |
| wavelet.HLH_glrlm_LongRunHighGrayLevelEmphasis.CT | 0.043598 |
| wavelet.LLH_gldm_DependenceVariance.CT | 0.043753 |
| wavelet.HHH_ngtdm_Contrast.CT | 0.04394 |
| original_glrlm_ShortRunLowGrayLevelEmphasis.PET | 0.044156 |
| log.sigma.3.0.mm.3D_gldm_SmallDependenceLowGrayLevelEmphasis.CT | 0.044216 |
| original_glrlm_LowGrayLevelRunEmphasis.PET | 0.044222 |
| original_gldm_LowGrayLevelEmphasis.PET | 0.044281 |
| wavelet.LHH_glrlm_RunLengthNonUniformityNormalized.CT | 0.044379 |
| wavelet.HHL_gldm_SmallDependenceHighGrayLevelEmphasis.PET | 0.044422 |
| original_glrlm_LongRunLowGrayLevelEmphasis.PET | 0.044487 |
| log.sigma.3.0.mm.3D_glszm_SmallAreaLowGrayLevelEmphasis.CT | 0.044716 |
| wavelet.LLL_glrlm_LowGrayLevelRunEmphasis.CT | 0.044748 |
| wavelet.LHL_glszm_LargeAreaLowGrayLevelEmphasis.PET | 0.044794 |
| wavelet.LHL_glszm_GrayLevelNonUniformity.CT | 0.044797 |
| log.sigma.2.0.mm.3D_glcm_Correlation.CT | 0.045057 |
| wavelet.LHL_glszm_SmallAreaLowGrayLevelEmphasis.CT | 0.04518 |
| wavelet.HLH_glrlm_RunPercentage.CT | 0.04523 |
| original_firstorder_10Percentile.PET | 0.045248 |
| wavelet.LHL_glrlm_RunPercentage.CT | 0.04534 |
| original_glcm_Imc1.PET | 0.045648 |
| log.sigma.3.0.mm.3D_gldm_LowGrayLevelEmphasis.CT | 0.045838 |
| wavelet.LLL_gldm_SmallDependenceLowGrayLevelEmphasis.CT | 0.046007 |
| wavelet.HHL_glszm_SmallAreaLowGrayLevelEmphasis.CT | 0.046057 |
| log.sigma.2.0.mm.3D_glrlm_RunLengthNonUniformity.PET | 0.046158 |
| wavelet.HHL_glrlm_RunLengthNonUniformityNormalized.CT | 0.046241 |
| wavelet.LHL_firstorder_Mean.PET | 0.04647 |
| wavelet.LLL_gldm_GrayLevelNonUniformity.CT | 0.046582 |
| log.sigma.3.0.mm.3D_glrlm_RunLengthNonUniformity.PET | 0.046729 |
| wavelet.LHL_glrlm_RunEntropy.CT | 0.046767 |
| wavelet.LLL_gldm_LowGrayLevelEmphasis.CT | 0.046891 |
| wavelet.LHL_glrlm_RunLengthNonUniformity.PET | 0.046917 |
| wavelet.HLL_gldm_SmallDependenceLowGrayLevelEmphasis.CT | 0.046948 |
| wavelet.LLH_firstorder_Range.CT | 0.046996 |
| wavelet.HHL_glszm_SmallAreaHighGrayLevelEmphasis.PET | 0.04705 |
| original_glszm_LargeAreaLowGrayLevelEmphasis.PET | 0.047053 |
| wavelet.LLH_firstorder_Uniformity.PET | 0.047085 |
| wavelet.LLH_glrlm_GrayLevelNonUniformityNormalized.PET | 0.047123 |
| wavelet.HLH_glszm_LowGrayLevelZoneEmphasis.PET | 0.047319 |
| wavelet.LLL_glszm_SmallAreaLowGrayLevelEmphasis.CT | 0.047657 |
| wavelet.HLL_glrlm_RunLengthNonUniformity.PET | 0.048513 |
| log.sigma.3.0.mm.3D_glrlm_ShortRunLowGrayLevelEmphasis.CT | 0.048568 |
| original_glszm_GrayLevelNonUniformityNormalized.PET | 0.048694 |
| wavelet.LHH_glszm_SizeZoneNonUniformity.CT | 0.0488 |
| original_glrlm_ShortRunHighGrayLevelEmphasis.CT | 0.048931 |
| wavelet.LLL_firstorder_10Percentile.PET | 0.048956 |
| wavelet.LHH_glcm_MCC.CT | 0.049001 |
| wavelet.LHL_glrlm_LongRunLowGrayLevelEmphasis.PET | 0.049039 |
| original_glrlm_GrayLevelNonUniformityNormalized.PET | 0.049113 |
| original_firstorder_Uniformity.PET | 0.049164 |
| wavelet.LLH_glszm_GrayLevelNonUniformityNormalized.PET | 0.049165 |
| wavelet.LHL_firstorder_Variance.PET | 0.049466 |
| wavelet.LHL_gldm_GrayLevelVariance.PET | 0.049506 |
| wavelet.LHL_glrlm_GrayLevelVariance.PET | 0.049554 |
| wavelet.LHL_gldm_LargeDependenceLowGrayLevelEmphasis.PET | 0.049765 |
| wavelet.HHL_glszm_HighGrayLevelZoneEmphasis.PET | 0.049807 |

**Supplementary Figure 1**

**Another two typical cases with DLBCL to support the clinical application of the nomogram.**


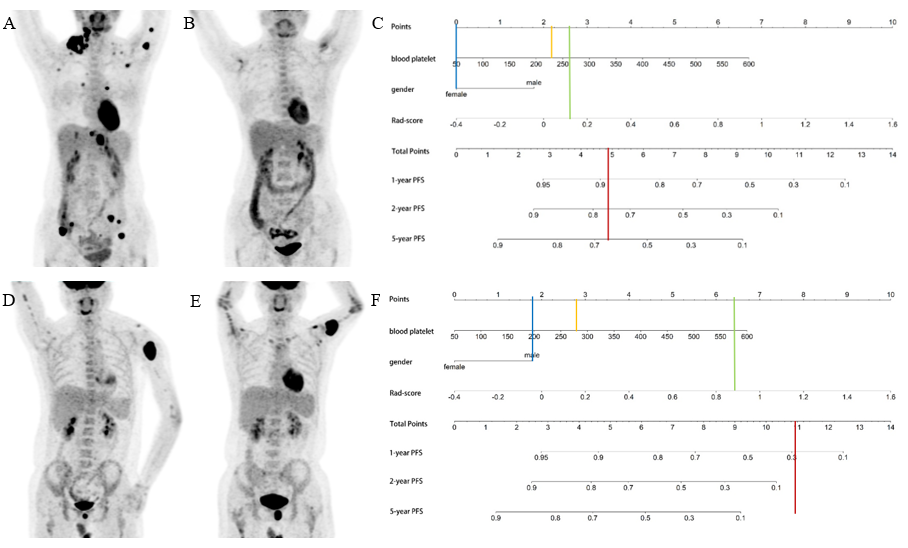


(A-C). Female, 52 years old, who underwent 6 cycles of R-CHOP regimen chemotherapy after firstly diagnosisd of DLBCL(A) and was confirmed as completely response(CR)(B). Blood platelet:228, the Rad-score: 0.11. Vertical lines of each variable were drawn(C) and total points: (0 + 2.2 + 2.65 = 4.85).

(D-F). Male, 35 years old, who underwent 4 cycles of R-CHOP regimen chemotherapy after firstly diagnosisd of DLBCL(D) and was confirmed as progression disease (PD) (E). Blood platelet:278, the Rad-score: 0.88. Vertical lines of each variable were drawn(F) and total points: (1.77 + 2.78 + 6.38 = 10.93).
